# Characterization of a Fetal Liver Cell Population Endowed with Long‐Term Multiorgan Endothelial Reconstitution Potential

**DOI:** 10.1002/stem.2494

**Published:** 2016-09-28

**Authors:** Ana Cañete, Valentine Comaills, Isabel Prados, Ana María Castro, Seddik Hammad, Patricia Ybot‐Gonzalez, Ernesto Bockamp, Jan G. Hengstler, Bertie Gottgens, María José Sánchez

**Affiliations:** ^1^ Centro Andaluz de Biología del Desarrollo (CABD)Consejo Superior de Investigaciones Científicas (CSIC), Junta de Andalucía (JA), Universidad Pablo de Olavide (UPO)SevillaSpain; ^2^ Faculty of Veterinary Medicine, Department of Forensic Medicine and Veterinary ToxicologySouth Valley UniversityQenaEgypt; ^3^ Leibniz Research Center for Working Environment and Human Factors (IfADo), TU Dortmund UniversityDortmundGermany; ^4^ Instituto de Biomedicina de Sevilla (IBIS), Hospital Universitario Virgen del Rocío, CSIC, Universidad de SevillaSevilleSpain; ^5^ Institute of Translational Immunology, University Medical Center, Johannes Gutenberg UniversityMainzGermany; ^6^ Cambridge Institute for Medical Research & Wellcome Trust and MRC Cambridge Stem Cell Institute, Cambridge UniversityUnited Kingdom

**Keywords:** Progenitor cells, Hematopoietic progenitors, Fetal liver, Endothelial reconstitution, Newborn transplantation

## Abstract

Stable reconstitution of vascular endothelial beds upon transplantation of progenitor cells represents an important challenge due to the paucity and generally limited integration/expansion potential of most identified vascular related cell subsets. We previously showed that mouse fetal liver (FL) hemato/vascular cells from day 12 of gestation (E12), expressing the Stem Cell Leukaemia (SCL) gene enhancer transgene (SCL‐PLAP^+^ cells), had robust endothelial engraftment potential when transferred to the blood stream of newborns or adult conditioned recipients, compared to the scarce vascular contribution of adult bone marrow cells. However, the specific SCL‐PLAP^+^ hematopoietic or endothelial cell subset responsible for the long‐term reconstituting endothelial cell (LTR‐EC) activity and its confinement to FL developmental stages remained unknown. Using a busulfan‐treated newborn transplantation model, we show that LTR‐EC activity is restricted to the SCL‐PLAP^+^VE‐cadherin^+^CD45^−^ cell population, devoid of hematopoietic reconstitution activity and largely composed by Lyve1^+^ endothelial‐committed cells. SCL‐PLAP^+^ Ve‐cadherin^+^CD45^−^ cells contributed to the liver sinusoidal endothelium and also to the heart, kidney and lung microvasculature. LTR‐EC activity was detected at different stages of FL development, yet marginal activity was identified in the adult liver, revealing unknown functional differences between fetal and adult liver endothelial/endothelial progenitors. Importantly, the observations that expanding donor‐derived vascular grafts colocalize with proliferating hepatocyte‐like cells and participate in the systemic circulation, support their functional integration into young livers. These findings offer new insights into the engraftment, phonotypical, and developmental characterization of a novel endothelial/endothelial progenitor cell subtype with multiorgan LTR‐EC activity, potentially instrumental for the treatment/genetic correction of vascular diseases. Stem Cells *2017;35:507–521*

**Significance Statement**

Using the newborn transplantation model, we have characterized in the mouse fetal liver a unique SCL‐PLAP^+^Ve‐cad^+^CD45^−^ population endowed with stable multiorgan endothelial reconstitution potential and mostly composed by endothelial committed cells. Considering clinical applications, transplantation of SCL‐PLAP^+^Ve‐cad^+^CD45^−^ cells may provide a more robust neonatal vascular engraftment than adult bone marrow‐derived or adult liver‐derived endothelial/endothelial progenitor cell populations, constituting a novel and highly promising source of cells to study vascular reconstitution and repair in neonatal preclinical models and also might help guide the derivation of long term reconstituting vascular progenitors from pluripotent stem cells.

## Introduction

Transplantation of vascular progenitor cells with potential to engraft and long‐term contribute to functional endothelial vascular beds in multiple organs has been proposed as an important strategy for genetic correction of vascular diseases, inducing vascular regeneration and as a tool for manipulation of vascular mediated organ homeostasis and growth 1, 2, 3. Evidence from several animal injury models demonstrated that infusion of primary and in vitro expanded postnatal circulating and bone marrow (BM)‐derived cells, selected by the expression of different endothelial and hematopoietic progenitor associated markers, including the Flk1^+^, VE‐cadherin^+^ Lin^−^ (VE‐cad^+^Lin^−^), CD31^+^, KIT^+^Sca^+^Lin^−^, KIT^+^CD34^+^Lin^−^ or CD133^+^CXCR4^+^ cell populations in the mouse 4, 5, 6, 7, 8, 9, promoted postnatal physiological and pathological neovascularization in different organs, including the liver 10, 11, 12, heart 13, 14, and kidney 15, 16, 17, 18. This has led to clinical trials of circulating/BM cell subsets for therapy of certain human vascular disorders 19. Apparently, infused cells participated in the recovery of injured vasculature through the production of paracrine factors that promote endogenous endothelial cell (EC) growth and/or by direct incorporation/differentiation into organ endothelial tubular structures. While paracrine neo‐angiogenic effects seem to be a BM‐derived cell‐mediated common mechanism involved in short term vascular injury recovery 6, 20, 21, 22, the long term incorporation potential of BM‐derived cell populations into organ vascular endothelium is considered highly variable, depending on the injured model, targeted organ or specific characteristics of the donor cell population 8, 23. Therefore, the search for cell sources with substantial vascular endothelial potential and functional organ integration capacity upon transplantation remains an important area of research to be explored.

Distinct subsets of postnatal primary microvascular ECs, able to stably reconstitute organ vascular beds upon transfer to injured tissue 24, 25 and in particular liver sinusoidal endothelial cells (LSECs), have been described. LSECs transferred to adult animals can efficiently integrate into and reconstitute the liver sinusoidal endothelium of acutely injured or regenerating livers 26, 27, 28, modulate liver regeneration 27, 29, fibrosis 30, 31, and alleviate coagulation defects 32. Despite the fact that transfer of LSECs constitute one of the most promising cell‐based strategies for restoration of vascular damage and organ homeostasis 33, 34, they display reduced plasticity when compared to circulating/BM‐derived endothelial progenitor cells (EPCs) and long term survival of the LSEC‐derived grafts is only observed in the liver 26, a factor that potentially restraints their use in applications involving systemic vascular pathologies.

Using intravenous cell transfer into myelo‐ablated newborn‐mice, we found that cells from the FL, a predominant hematopoietic organ at this stage, had a much superior contribution potential to the endothelium of different organs than postnatal BM cells 35. Newborns present an active organ growth and neovascularization activity, thereby it was proposed that this might facilitate the engraftment of transferred EC/EPCs in different locations 35, 36. The FL multiorgan EC engraftment potential is hence referred as long‐term reconstituting EC (LTR‐EC) activity, involving cell homing/lodging, differentiation to organ‐associated endothelial types and long term stabilization of the donor‐derived vascular beds. In the current study, we determine the hematopoietic and endothelial reconstitution potential of different E12 FL SCL‐PLAP^+^ cell subsets, characterized by the activation of the hematopoietic stem cell/endothelial SCL‐3′Enhancer, derived from the stem cell leukaemia (SCL) gene, a key transcription factor for the normal development of hematopoietic progenitors and blood vessels 37, 38, 39, 40. We isolate and characterize a novel progenitor population that is strongly committed to the endothelial lineage, likely responsible for the LTR‐EC activity at different stages of FL development but functionally absent in the adult liver. Furthermore, productive integration of the grafted vasculature is supported by its association to proliferating hepatocyte‐like foci together with participation in the general circulation. To our knowledge, the here described FL cell population represents a so far not recognized largely committed EC/EPC cell subset endowed with long‐term and multiorgan vascular reconstituting potential.

## Materials and Methods

### Mice

Animals were maintained in the CABD animal care facility and procedures performed under the guidance of the European Community Legislation with the approval of ethical committee of CSIC and Universidad Pablo de Olavide. Transgenic lines for transplantation assays include the SCL3′Enh‐hPLAP mice, expressing the human placental alkaline phosphatase (PLAP) reporter gene 35, 39, the SCL‐3′Enh‐Lacz mice expressing the β‐galactosidase reporter gene 41 and the actin‐DsRed mice expressing the DsRed‐MST reporter gene under the actin regulatory elements (Jackson Laboratories, stock 005441) 42.

### Cell Preparation

To obtain fetal tissues, timed breeding of heterozygous/homozygous SCL‐3′Enh‐PLAP transgenic mice was established. Fetuses were obtained from day 10 (E10) to day 14 (E14) of gestation. Cell preparation procedures from embryonic/fetal and adult hematopoietic tissues were previously published 35, 43, (Supporting Information). A fragment of yolk sac (YS) was used for embryo typing by staining with the PLAP substrate, nitroblue tetrazolium (NBT) (Roche, Mannheim, Germany, www.lifescience.roche.com) as described 39. LSEC enriched nonparenchymal cell (NPC) fraction was obtained from adult mouse livers by a two‐step collagenase perfusion technique and gradient percoll centrifugation as described 26, 29 with modifications (Supporting Information).

### Flow Cytometry Analysis and Cell Sorting

Cell suspensions were stained, analyzed and separated by flow cytometry as previously described 35 (Sup. Information). A FACSAria flow cytometer equipped with two lasers and run with a FACSDiva software (BD Biosciences, www.bdbiosciences.com/eu) was used.

### Newborn Transplantation Assay and Chimerism Analysis

Transplantation assays were performed transferring cells to the facial vein of wild type busulfan treated newborn mice as described in detail 35, 44, 45 (Supporting Information). Long‐term hematopoietic and vascular reconstitution analysis was performed at 3‐8 months post‐transplant. Mice were anesthetized, blood collected from the heart and perfused with 50 ml Tris‐buffered saline containing 0.001% heparin. Hematopoietic organs were homogenized in phosphate buffered saline (PBS) supplemented with 5% of foetal calf serum (FCS) (PBS 5%FCS). The liver, heart, kidney and lung were fixed in zinc solution (BD‐Pharmingen, San Diego, CA, www.bdbioscience.com). For studies related to immuno‐histological detection of proliferative markers and DsRed reporter gene, mice were perfused with PBS and the liver fixed in 4% formaldehyde solutions (Merck KGaA, Germany). Perfusion was not performed on <4*–*weeks*–*old mice and when livers were weighed.

Hematopoietic engraftment was assessed by PCR for PLAP and LacZ reporter genes on genomic DNA from peripheral blood and hematopoietic organs and by FACS for PLAP and CD45 expression in peripheral blood 35. Mice were considered reconstituted when circulating SCL‐PLAP^+^ cells represented at least 1%. Levels of hematopoietic chimerism were also determined by semi‐quantitative PCR for PLAP and LacZ on genomic DNA from hematopoietic organs as published 35, 43 (Supporting Information). In some experiments, the DsRed marker was used for multilineage hematopoietic contribution.

Vascular engraftment was assessed by systematic histological screening for SCL‐PLAP^+^ donor‐derived sinusoidal vascular‐like clusters (v.c.) on liver sections by NBT staining 35 (Supporitng Information). For quantification of vascular engraftment on NBT stained liver sections we determined the frequency of mice positive for v.c. and the relative tissue area containing v.c., denominated vascular cluster area (v.c.a.), using the Image AnalySIS software program V3.1.110, as detailed in Supporting Information Figure 1C and Supporting Information. Heart, kidney, and lung sections from selected mice were also screened by NBT staining and scored as indicated in Supporting Information Table 1. Triple immunostaining for PLAP, CD45 and Isolectin B4 (IsoB4) or CD31 was performed to confirm the endothelial nature of the v.c. as described 17, 35. Costaining for Ki67, P‐H3 and albumin was assessed on 4% formaldehyde fixed liver sections from some 3 weeks old mice. Detection of DsRed fluorescence and PLAP immunostaining was performed on cryosections. For expanded immune‐histological reagents, antibodies, and methods refer to Supporting Information.

### OP9 Endothelial Cord/Colony Forming Assay

OP9 stromal cell line, expressing GFP, was provided by J.C. Zuñiga‐Pflucker 46. Cells were cocultured in 96‐well plates containing subconfluent OP9 cells in MEMα containing 10% FCS, 1% P/S, 5×10^−5^ M 2β−mercaptoethanol (Sigma‐Aldrich, Steinheim, Germany, www.sigmaaldrich.com) and 50 ng/ml VEGF (Cell Signaling Technology, www.cellsignal.com) 47, 48. After 4 days, cells from duplicated wells were fixed in Methanol/DMSO 5% and stained for CD31 as described with modifications 47 (Supporting Information). Sequential images covering the area of each well were generated and single CD31^+^ cords and colonies counted. Images were obtained with a Leica DMIRB inverted microscope including a Leica DFC350FX digital camera (www.leica-microsystems.com).

### Hematopoietic CFU‐C Assay

Clonogenic Colony Forming Unit in Culture (CFU‐C) for erythroid/myeloid progenitor assay was performed as described 40. In brief, FL sorted cells were transferred to 1.5 ml of cytokine‐supplemented methylcellulose medium (M3434, StemCell Technologies, www.stemcell.com) and plated in duplicates in 6‐well plates. Hematopoietic colonies were scored after 7 days under a Leica MZ7.5 stereoscope including a Nikon DS5 digital camera and DSL1 control unit (http://www.nikon.es/es_ES/).

### B Lymphoid Culture Assay

OP9 B lymphoid differentiation/expansion assay was performed as described 49, 50 with slight modifications. FL sorted cells were cultured for 14 days on confluent OP9 layers in 24‐well plates in supplemented MEMα containing IL‐7 (50 U/ml) and Flt3 ligand (10 ng/ml) (PetroTech, Rocky Hill, NJ, www.peprotech.com). Cells were re‐fed every 4 days and replated once onto a new well with OP9 cells. Cells were analyzed by flow cytometry gating out 7AAD^+^ dead cells and GFP^+^ OP9 cells.

### BrdU Treatment, Immuno‐Detection and Quantification

Mice received intraperitoneal (i.p.) injections of 5‐Bromo‐2‐DeoxyUridine (BrdU) (Sigma‐Aldrich, Stenheim, Germany, www.sigmaaldrich.com) (80 mg/kg body weight in 0.9% NaCl solution) 4 hours before analysis as described 51. Vibratome thick liver sections were immunostained for detection of BrdU, PLAP and nuclei as described 51 (Supporting Information). *Z*‐stack images were taken with a Leica SP5 confocal MP‐AOBS microscope (60‐80 optical slices of 0.5 μm depth). The number of total nuclei and outline of tissue area per image was determined using the Imaris software (Version 7.6.3).

### Statistical Analysis

Student's *t* test was used to compare mean ± SD from two groups with parametric distribution. Comparison for donor‐derived vascular cluster area (v.c.a.) at different times post‐transplantation was evaluated using a nonparametric U‐Mann–Whitney test. Statistical significance was defined as *p* < .05. Excel 14.3.4 and IBM‐PSS Statistics 19 software were used.

## Results

### Multiorgan Long Term Reconstituting Endothelial Cell Activity Is Identified in the SCL‐PLAP^+^VE‐cad^+^CD45^−^ Cell Subset from E12 FL

**Figure 1:**
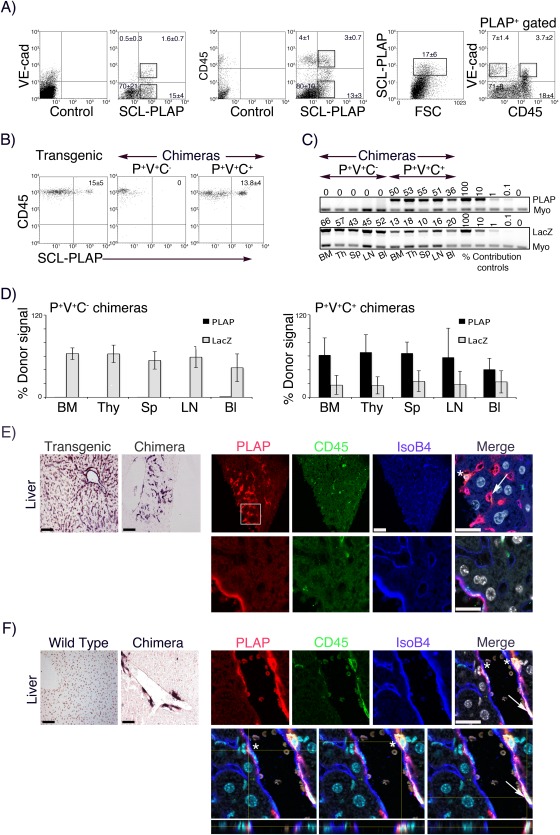
Long‐term reconstituting endothelial cell activity is identified in the SCL‐PLAP^+^VE‐cad^+^CD45^−^ cell subset from E12 FL. Cell suspension was prepared from E12 FL SCL‐3′Enh‐PLAP transgenics. **(A)**: FACS profiles showing representative sorting windows according to the expression of SCL‐3′Enh‐PLAP (P^+^) transgene and VE‐cad (V^+^), SCL‐PLAP and CD45 (C^+^) and SCL‐PLAP, VE‐cad and CD45. Quadrants are set according to values obtained from unstained and single stained controls (left plots on P/V and P/C staining) on viable cells (11 ± 5% 7AAD^+^ dead cells). Percentage ±SD of cells in each quadrant and SCL‐PLAP^bright+^ gated cells are indicated (*n* = 13). **(B)**: Representative FACS plots showing percentage ± SD of SCL‐PLAP^+^CD45^+^ hematopoietic cells in peripheral blood from SCL‐3′Enh‐PLAP transgenics (*n* = 7) and transplanted mice with P^+^V^+^C^‐^ (*n* = 9) and P^+^V^+^C^+^ cells (*n* = 7). Results from three independent transplantation experiments. **(C)**: Semiquantitative PCR on genomic DNA from hematopoietic organs from representative P^+^V^+^C^+^ and P^+^V^+^C^−^ transplanted mice showing the levels of the donor markers PLAP and LacZ transgenes (from cotransplanted BM‐LacZ cells). Numbers in PCR gel images indicate the percentage of donor‐marker contribution calculated by comparison with the control signal curve using Myo gene as normalization control. **(D)**: Percentage of donor cell chimerism in different hematopoietic organs (P^+^V^+^C^−^ chimeras, *n* = 4 from two independent transplantation experiments; P^+^V^+^C^+^ chimeras, *n* = 6 from three independent transplantation experiments). (BM, bone marrow; Thy, thymus; Sp, spleen; LN, lymph nodes; Bl, blood). **(E)**: Representative liver sections from an SCL‐3′Enh‐PLAP transgenic mouse and a P^+^V^+^C^−^ chimera stained with nitroblue tetrazolium (NBT) (left images) and with antibodies for detection of PLAP, blood cells (CD45), endothelial cells (IsoB4) and nuclei (DAPI). P^+^V^+^C^−^ cell contributed to sinusoidal vascular clusters containing SCL‐PLAP^+^CD45^−^IsoB4^+^ endothelial (arrows) and sporadic SCL‐PLAP^+^CD45^+^ blood cells (asterisks) (top images) and to the endothelium of large vessels (lower images). **(F)**: Liver sections from wild type and a P^+^V^+^C^+^ chimera showing PLAP^+^ donor cells mostly in perivascular locations (top images). Z‐stack images and orthogonal projections showing SCL‐PLAP^+^CD45^+^IsoB4^−^ cells (asterisk) located behind the IsoB4^+^ endothelium (blue) and an SCL‐PLAP^+^CD45^+^IsoB4^+^ cell (arrow) within the endothelium (Z‐stacks from six optical images acquired at 2 μm intervals). Scale bars NBT images 100 μm. Scale bars immunofluorescence images 25 μm. Abbreviations: FSC, Forward Side Scatter; PLAP, placental alkaline phosphatase reporter gene; SCL, stem cell leukaemia gene.

FL cells expressing high levels of the SCL‐3′Enh‐PLAP reporter transgene (SCL‐PLAP^+^ cells) presented long‐term hematopoietic and endothelial reconstitution activity upon transplantation 35, 39. To determine whether long‐term endothelial reconstitution (LTR‐EC) activity was associated to a specific SCL‐PLAP^+^ cell subset, cells were FACS sorted, i.v. transplanted to busulfan conditioned newborn recipient mice and hematopoietic and endothelial contribution analyzed at >3 months. SCL‐PLAP^+^ FL cells were fractionated based on the surface expression of the EC receptor VE‐cadherin (VE‐cad), expressed in the embryonic hemangioblasts 52, postnatal subsets of EPCs 5 and hematopoietic stem and progenitor cells (HSPCs) 47, 53, 54 and the pan‐leukocyte marker CD45, (Fig. 1A). Long‐term engraftment analysis showed that most animals transplanted with FL SCL‐PLAP^+^VE‐cad^+^ or SCL‐PLAP^+^VE‐cad^−^ cells presented donor‐derived hematopoietic chimerism in peripheral blood as determined by FACS detection of the donor marker PLAP and in hematopoietic organs by PCR‐PLAP signal on genomic DNA, (Table 1, Supporting Information Fig. 1A‐C), consistent with previous reports 47. However, while SCL‐PLAP^+^VE‐cad^+^ cells contributed to NBT‐positive liver sinusoidal endothelial vascular‐like clusters (v.c.) and to some endothelial‐like cells in large vessels (Table 1, Supporting Information Fig. 1D), only few nonendothelial‐like SCL‐PLAP^+^VE‐cad^−^ derived cells were observed in liver sections. Donor‐derived ECs identity was then confirmed by the expression of the EC marker IsoB4 and the absence of the hematopoietic marker CD45 35, 55 (Supporting Information Fig. 1E). FL SCL‐PLAP^+^ cells subdivision based on expression of CD45 (Fig. 1A), showed that donor‐derived sinusoidal v.c. were only observed in animals transferred with SCL‐PLAP^+^CD45^−^ cells and no contribution to liver ECs was detected in SCL‐PLAP^+^CD45^+^ hematopoietic chimeras (Table 1). Further transfer of FL SCL‐PLAP^+^VE‐cad^+^CD45^−^ and SCL‐PLAP^+^VE‐cad^+^CD45^+^ cells (Fig. 1A), revealed that donor‐derived liver sinusoidal v.c. and ECs within large vessels were limited to mice transferred with the SCL‐PLAP^+^VE‐cad^+^CD45^−^ population while hematopoietic reconstitution activity was present in SCL‐PLAP^+^VE‐cad^+^CD45^+^ cells (Table 1, Fig. 1B‐E). Of note, although FL SCL‐PLAP^+^VE‐cad^+^CD45^−^ cells did not present hematopoietic reconstitution potential (Fig. 1B‐D), sporadic donor‐derived CD45^+^ hematopoietic cells were detected within the v.c. (Fig. 1E). Overall, endothelial contribution analysis in the liver indicated that LTR‐EC activity was restricted to FL SCL‐PLAP^+^VE‐cad^+^CD45^−^ cells.

**Table 1:** Hematopoietic and liver vascular engraftment potential of E12 FL SCL‐PLAP^+^ cell populations

Donor cells		Hematopoietic chimerism		Vascular chimerism	
Population	Cell nº × 10^4^ (ee)	nº PLAP^+^/ Total mice^a^	% PLAP^+^ (Range values)^b^	nº PLAP^+^/ Total mice^c^ (%)	PLAP^+^ v.c.a. (×10^−3^ cm^2^) (Range values)
VE‐cad^+^	1‐3	8/12	7.7 ± 4.4	6/8	0.42 ± 0.44
	(3‐8)		(1.4‐14)	(75)	(0.004‐1.05)
VE‐cad^‐^	5‐15	7/8	9.8 ± 8	0/7	0
	(2‐8)		(1‐22)	(0)	
CD45^+^	2‐8	19/27	7.6 ± 4.5	0/19	0
	(3‐5)		(1.7‐15)	(0)	
CD45^‐^	2‐5	0/17	0	3/13	0.15 ± 0.05
	(3)			(23)	(0.09‐0.19)
VE‐cad^+^CD45^+^	1‐2	9/14	13.8 ± 3.8	0/9	0
	(4‐6)		(8‐19)	(0)	
VE‐cad^+^CD45^‐^	1‐2	0/9	0	3/9	10 ± 14
	(3‐6)			(33)	(0.18‐4.7‐27)

Mice were i.v. transferred with the indicated number of FL sorted SCL‐PLAP^+^ populations plus 1 x 10^6^ BM‐LacZ^+^ cells and analysed at 3‐8 months post‐transplant for hematopoietic and vascular engraftment. SCL‐PLAP^+^ hematopoietic chimerism was determined in peripheral blood leukocytes by PCR‐PLAP and flow cytometry.

a Only mice presenting PLAP and/or LacZ PCR signal in circulation are included in the study. The number of PLAP^+^ chimeras related to the total analysed mice is indicated.

b Mean values for the % of SCL‐PLAP^+^CD45^+^ leukocytes in the host circulation assessed by flow cytometry. All hematopoietic chimeras were positive for PCR‐PLAP signal in hematopoietic organs. Significant increment on long‐term circulating donor‐derived cells were obtained from SCL‐PLAP^+^VE‐cad^+^CD45^+^ chimeras compared to SCL‐PLAP^+^VE‐cad^+^ and SCL‐PLAP^+^CD45^+^ chimeras (*p* < ,05, Student's *t* test), potentially reflecting an enrichment on HSCs. Vascular chimerism was determined by histological NBT detection of SCL‐PLAP^+^ donor‐derived cells forming vascular‐like clusters (v.c.) on liver sections (Supporting Information Fig. 1D) and animals scored as positive when at least one v.c. was observed.

c The number and percentage of animals presenting SCL‐PLAP^+^ v.c. in liver sections from the total number of SCL‐PLAP^+^ hematopoietic chimeras is shown, except for mice transplanted with SCL‐PLAP^+^CD45^‐^ and SCL‐PLAP^+^VE‐cad^+^CD45^‐^ cells that did not present FL derived hematopoietic engraftment in circulation, (see Supporting Information Table 1 for individual values). The mean values of tissue area containing SCL‐PLAP^+^ v.c. referred to the total tissue area analysed are indicated for each group, (PLAP^+^ v.c.a.). The mean ± SD and range values obtained from vascular chimeras from each group are shown (see Supporting Information Table 1 for individual values). Data was obtained from 3 to 6 independent transplantation experiments for each population.

Abbreviations: ee, embryo equivalent; PLAP, placental alkaline phosphatase reporter gene.

Considering the reported endothelial‐like phenotype of the VE‐cad^+^CD45^+^ embryonic population endowed with HSPC activity 47, 53, we looked in detail for donor‐derived ECs in SCL‐PLAP^+^VE‐cad^+^CD45^+^ chimeras. Z‐stack high resolution confocal microscopy images from 20 individual SCL‐PLAP^+^VE‐cad^+^CD45^+^‐derived PLAP^+^ cells, placed within the intima layer in large vessels, showed that only 7 cells located in an endothelial position facing the lumen and having a SCL‐PLAP^+^CD45^+^IsoB^+^ hematopoietic/endothelial mix phenotype. All other cells were peri‐endothelial SCL‐PLAP^+^CD45^+^IsoB^−^ hematopoietic cells (Fig. 1F, Supporting Information Table 2). Similar to long‐term transplanted primary leukemia cells that incorporated into the liver vascular endothelium as CD45^+^ endothelial‐like cells 56, this result suggested that rare hematopoietic committed cells were able to preserve hematopoietic features upon endothelial integration. Nevertheless, we cannot exclude the possibility that FL SCL‐PLAP^+^VE‐cad^+^CD45^+^ cells may contribute to CD45^−^ ECs in other models of acute vascular damage, including retinal ischemia or aggressive lung tumor models, as shown for transplanted adult BM‐derived HSCs/progeny or myeloid progenitors 7, 8.

**Figure 2:**
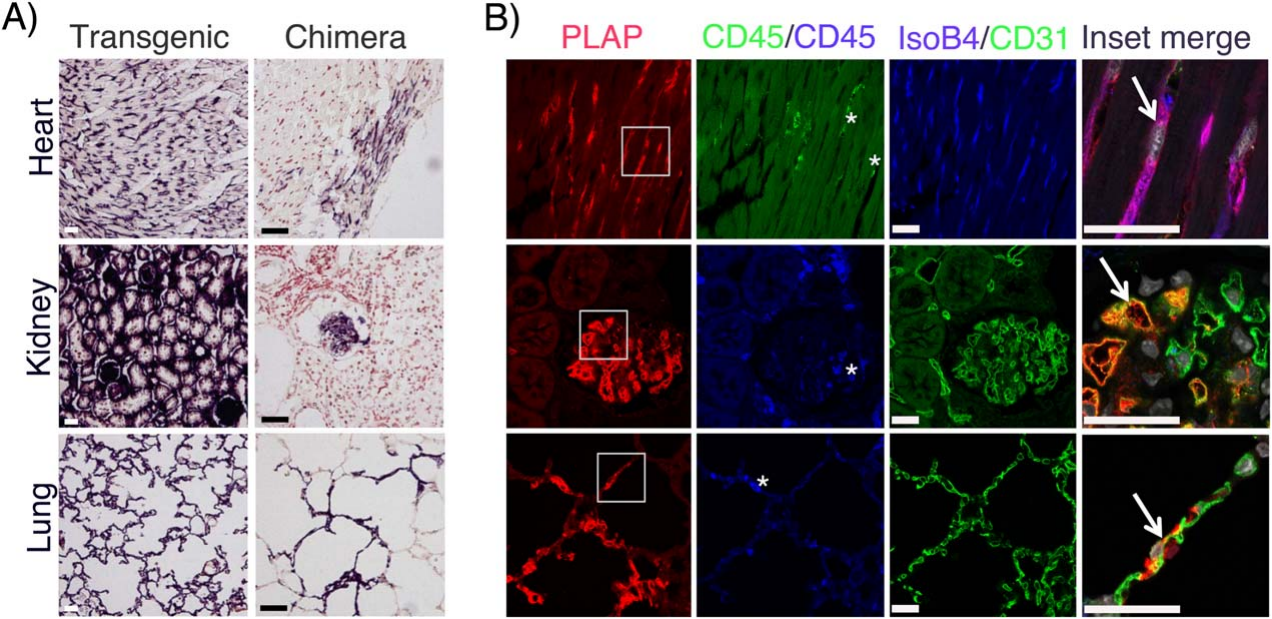
SCL‐PLAP^+^VE‐cad^+^CD45^−^ cells present long‐term reconstitution endothelial cell activity in different organs. SCL‐PLAP^+^VE‐cad^+^CD45^−^ chimeras were analyzed for endothelial engraftment in different organs. **(A)**: NBT staining on tissue sections obtained from indicated organs from a SCL‐3'Enh‐PLAP transgenic mouse and from a representative SCL‐PLAP^+^VE‐cad^+^CD45^−^ chimera. **(B)**: Images from stained sections with antibodies anti‐PLAP, anti‐CD45, and IsoB4/anti‐CD31 and DAPI (nuclei). Most donor‐derived cells are PLAP^+^CD45^‐^IsoB4^+^/CD31^+^ endothelial cells (arrows). Sporadic SCL‐PLAP^+^CD45^+^ hematopoietic cells are also detected (asterisk). Mice analyzed, *n* = 9. Heart and kidney endothelial contribution was observed in 4 and 3 mice, respectively; lung endothelial contribution was observed in 3 mice. Scale bars 25 μm. Abbreviation: PLAP, placental alkaline phosphatase reporter gene.

We next analyzed the long‐term endothelial contribution in hearts and kidneys from selected chimeric mice, (Supporting Information Table 1). Endothelial vascular clusters in the heart were only observed in mice transferred with SCL‐PLAP^+^VE‐cad^+^ (2 out of 6 mice presenting liver sinusoidal v.c.) (Supporting Information Fig. 1E) and SCL‐PLAP^+^VE‐cad^+^CD45^−^ cells (3 out of 9 analyzed mice, 2 of them presenting liver sinusoidal v.c.), (Fig. 2). Detection of donor cells in the kidneys was highly variable in mice presenting hematopoietic chimerism (SCL‐PLAP^+^VE‐cad^+^, SCL‐PLAP^+^VE‐cad^−^, SCL‐PLAP^+^CD45^+^ and SCL‐PLAP^+^VE‐cad^+^CD45^+^ chimeras), with consistent detection of scattered and/or large clusters of donor‐derived SCL‐PLAP^+^CD45^+^ hematopoietic cells and total absence of endothelial contribution (Supporting Information Figs. 1E, 2). Donor‐derived kidney ECs were only detected in SCL‐PLAP^+^CD45^−^ (in 2 out of 3 mice presenting liver sinusoidal v.c.) (Supporting Information Fig. 2) and SCL‐PLAP^+^VE‐cad^+^CD45^−^ chimeras (in 3 out of 9 recipient mice) (Fig. 2). Importantly, we also found an entire donor‐derived nephron vascular network, indicating that SCL‐PLAP^+^VE‐cad^+^CD45^−^ cells were capable of generating highly specialized ECs (Fig. 2). Furthermore, transplanted SCL‐PLAP^+^VE‐cad^+^CD45^−^ cells also formed vascular clusters in the lungs from all thee liver chimeras (Fig. 2). Overall, our results directly demonstrate that FL SCL‐PLAP^+^VE‐cad^+^CD45^−^ cells have multiorgan LTR‐EC potential.

### Most E12 FL SCL‐PLAP^+^VE‐cad^+^CD45^−^ Cells are Lyve1^+^ Endothelial Cells

**Figure 3:**
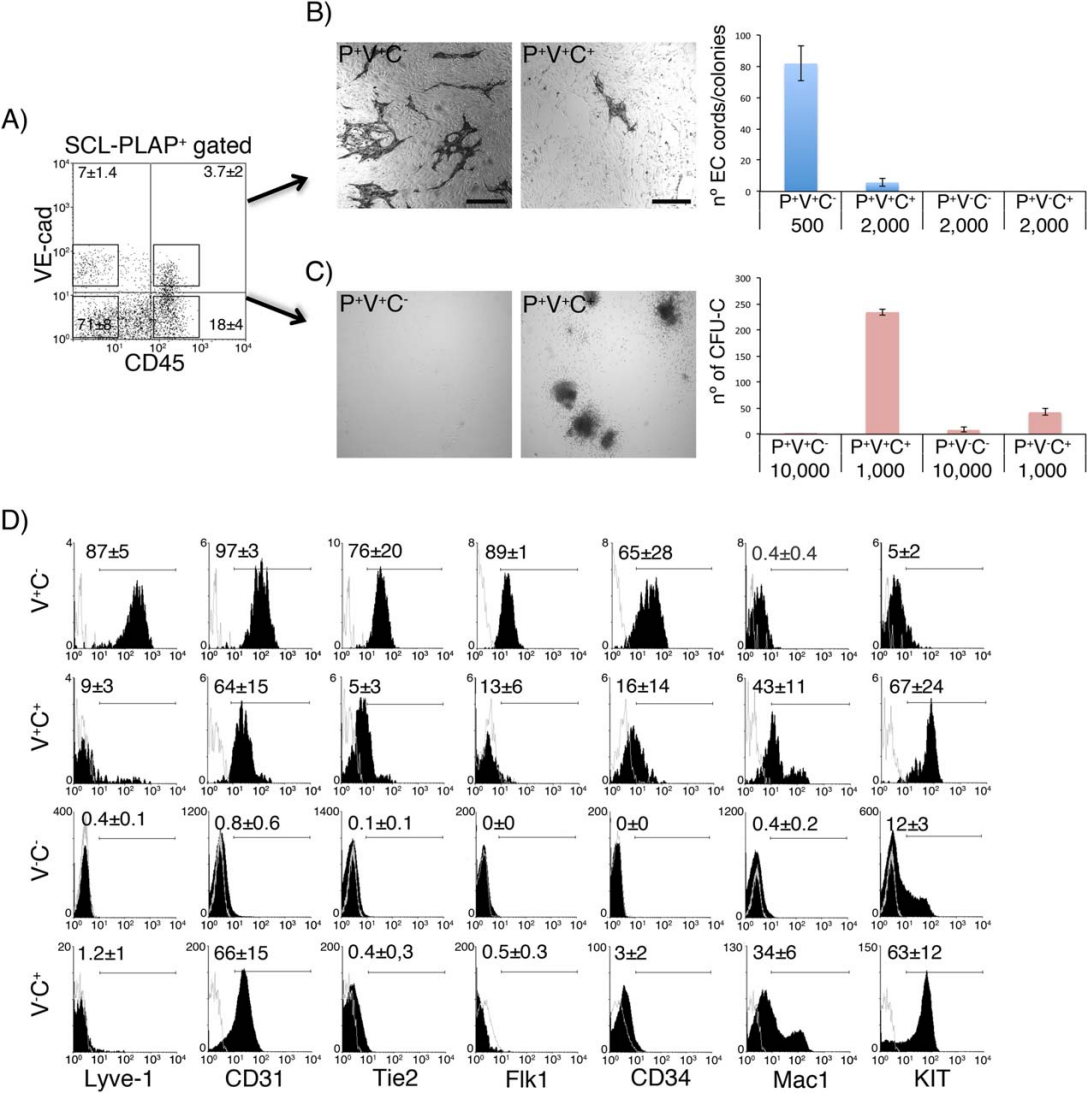
E12 FL SCL‐PLAP^+^VE‐cad^+^CD45^−^ population is largely composed by endothelial lineage committed Lyve1^+^ cells**. (A)**: FL cell fractions were separated by FACS according to the indicated representative sorting windows and in vitro endothelial and hematopoietic potential determined. **(B)**: Endothelial assays. Sorted cells were cultured on OP9 feeder cells for 4 days, stained with anti‐CD31 antibody and the number of CD31^+^ EC cord/colonies enumerated. Representative images from wells plated with 500 P^+^V^+^C^‐^ cells and 2,000 P^+^V^+^C^+^ cells are shown. The mean colony number ±SD per well plated with the indicated cell type and number is shown. EC activity was largely restricted to P^+^V^+^C^−^ cells, a mean value of 80 CD31^+^ EC cords/colonies were produced by 500 P^+^V^+^C^‐^ cells and extensive EC network by 2,000 cells (not shown). The number of scored wells was as follows: P^+^V^+^C^−^, *n* = 9; P^+^V^+^C^+^, *n* = 4; P^+^V^‐^C^−^, *n* = 5; P^+^V^−^C^+^, *n* = 5. Data collected from at least 2 independent experiments. **(C)**: CFU‐C myeloid/erythroid colony assays. Representative images from wells plated with 10^4^ P^+^V^+^C^‐^ and 10^3^ P^+^V^+^C^+^ cells cultured for 7 days. P^+^V^+^C^−^ cells did not present clonogenic CFU‐C hematopoietic progenitor potential. The mean colony number ±SD per well is shown. The number of scored wells was as follows: P^+^V^+^C^−^, *n* = 6; P^+^V^+^C^+^, *n* = 6; P^+^V^‐^C^−^, *n* = 6; P^+^V^‐^C^+^, *n* = 4. Data collected from three independent cell culture experiments. **(D)**: Flow cytometry analysis of viable FL populations subdivided according to the expression of VE‐cad (V) and CD45 (C). VE‐cad^+^ cells represent a subpopulation of SCL‐PLAP^+^ cells, (see Figure 1A). Representative histograms including unstained control (gray line) and specific antibody staining (black) are presented. Higher levels of endothelial markers (Lyve1, CD31, Tie2, Flk1, CD34), lack of expression of Mac1 and lower expression of progenitor KIT marker indicate a predominant endothelial lineage of SCL‐PLAP^+^VE‐cad^+^CD45^−^ cells. The percentages ± SD of positive cells are indicated. Three to four experiments for each marker were performed. Abbreviations: FL, Fetal liver; PLAP, placental alkaline phosphatase reporter gene; SCL, stem cell leukaemia gene.

To further analyze the endothelial/hematopoietic lineage commitment of the SCL‐PLAP^+^VE‐cad^+^CD45^−^ population, limiting dilution cultures assays for endothelial and hematopoietic progenitor cells were performed. Endothelial potential was assessed by the OP9/VEGF endothelial cord/colony formation assay, used to test EC differentiation/growth activity from embryonic/fetal cell subsets 47, 48. SCL‐PLAP^+^VE‐cad^+^CD45^−^ cells generated CD31‐positive ECs cords/colonies (80 ± 12 colonies per 500 cells) whereas EC activity was almost absent from other SCL‐PLAP^+^ populations (Fig. 3A, 3B). Also, SCL‐PLAP^+^VE‐cad^+^CD45^−^ cells were largely devoid of clonogenic myeloid/erythroid progenitor CFU‐C activity (Fig. 3C), supporting their commitment to the endothelial lineage observed in vivo.

Next, we performed a phenotypic characterization and determined the expression of endothelial and hematopoietic markers. As the vast majority of VE‐cad^+^ cells were included within the SCL‐PLAP^+^ population (Fig. 1A), the phenotype of the VE‐cad^+^ subsets was considered as a bona fide representation of the SCL‐PLAP^+^VE‐cad^+^ phenotype. FACS analysis revealed that most VE‐cad^+^CD45^−^ cells (87 ± 5%) expressed Lyve1, a marker for FL sinusoidal endothelium 57, 58, also present in a minor fraction of VE‐cad^+^CD45^+^ cells potentially representing pro‐angiogenic myeloid cells 59 (Fig. 3D). The presence of other pan‐endothelial related markers (CD31, Tie2, Flk1, and CD34) and the absence of the hematopoietic receptor Mac‐1, further reinforced the endothelial nature of SCL‐PLAP^+^VE‐cad^+^CD45^−^ cells (Fig. 3D). Very few KIT^+^ cells (5 ± 2%) were found within the VE‐cad^+^CD45^−^ population (Fig. 3D), a marker associated with embryonic VE‐cad^+^ hemogenic endothelium 49 and early HSPCs 50, 60, 61. Indeed, cultured VE‐cad^+^CD45^−^KIT^+^ cells showed some CD19^+^ B cells activity (Supporting Information Fig. 3). Together, in vitro differentiation and phenotypic analysis indicated that the SCL‐PLAP^+^VE‐cad^+^CD45^−^ population is mostly composed by endothelial committed Lyve1^+^ cells and unveiled its limited hematopoietic potential.

### LTR‐EC Activity is Largely Ascribed to Fetal Liver Developmental Stages

**Figure 4:**
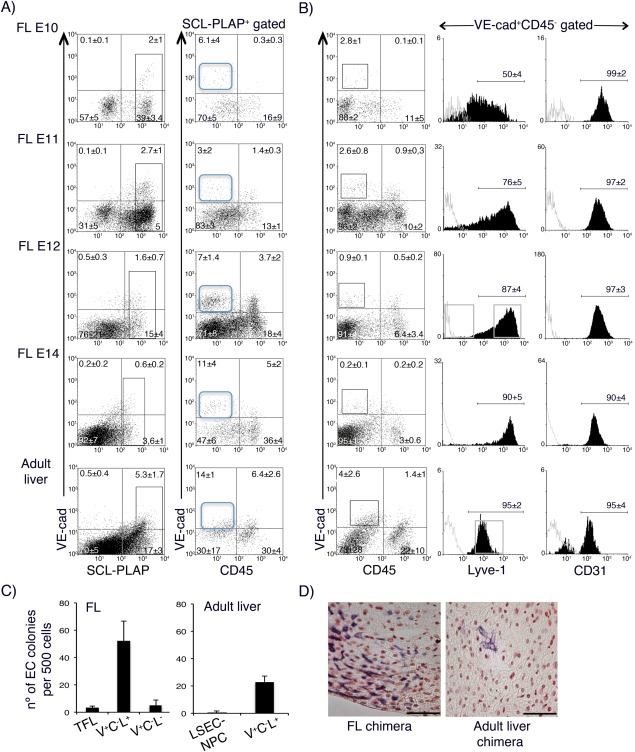
Characterization of SCL‐PLAP^+^VE‐cad^+^CD45^−^ cells during liver development and adult liver. FL cells from indicated embryonic stages and adult liver LSEC‐NPC fraction were obtained from SCL‐3'Enh‐PLAP transgenics. **(A)**: FACS analysis for SCL‐PLAP, VE‐cad and CD45 co‐expression. SCL‐PLAP^bright+^ cells are gated (left panels) and the percentage of VE‐cad^+^CD45^−^ determined at each developmental stage. SCL‐PLAP^+^VE‐cad^+^CD45^−^ cells are detected during development and in adult liver (blue gate). Cell suspensions from pooled E10‐E12 FL and from individual E14 FL and adult livers were analyzed. E10, *n* = 4; E11, *n* = 6; E12, *n* = 13; E14, *n* = 12; adults, *n* = 4. **(B)**: VE‐cad^+^CD45^−^ profile for Lyve1 and CD31 endothelial markers expression. Most VE‐cad^+^CD45^−^ express CD31 while the Lyve1 levels increment with development. Overlaid gray line histograms correspond to unstained controls on VE‐cad^+^CD45^−^ gated cells. E10, *n* = 3; E11, *n* = 6; E12, *n* = 6; E14, *n* = 8; adults, *n* = 3. Percentages ±SD of positive cells are shown. 7AAD^+^ dead cells were gated out from the analysis window and they represented 12 ± 4% in FL E10, 8 ± 5% in FL E11, 11 ± 5% in FL E12, 5 ± 2% in E14 FL and 68 ± 17% in adult liver LSEC‐NPC fraction. Embryonic/fetal tissues were obtained from at least 3 independent litters for each stage. **(C)**: The number of CD31^+^ EC cords/colonies from 500 plated unfractionated and sorted cell populations from E12 FL and adult liver LSEC‐NPC fraction is shown (representative sorting windows shown in B). Incremented EC activity is observed from total (TFL) and sorted VE‐cad^+^CD45^‐^Lyve1^+^ (V^+^C^−^L^+^) FL cells compared to adult liver‐derived counterpart populations. Very low EC activity is identified in FL VE‐cad^+^CD45^‐^Lyve1^−^ (V^+^C^−^L^−^) (5 ± 3 colonies) compared to V^+^C^−^L^+^ cells (52 ± 14 colonies). The number of scored wells was as follows: TFL, *n* = 5; FL V^+^C^−^L^+^, *n* = 9; FL V^+^C^−^L^−^, *n* = 9; adult liver LSEC‐NPC, *n* = 5; adult liver V^+^C^−^L^+^, *n* = 6. Two to six independent experiments, including simultaneous cultures for FL and adult liver cells, were performed. **(D)**: Images from NBT staining on liver sections from E11 FL chimera, showing part of a large PLAP^+^ vascular‐like cluster, and an adult liver chimera, transplanted with LSEC‐NPC cells, showing PLAP^+^ sporadic sinusoidal‐like cells. Number of adult liver chimeras analyzed, *n* = 6; sporadic sinusoidal‐like cells were observed in two animals. E11 FL chimeras, *n* = 6, large vascular clusters observed in all chimeras (see Table 2 and Supporting Information Table 4). Scale bars 100 μm. Abbreviations: LSECs, liver sinusoidal endothelial cells; NPC, nonparenchymal cell; PLAP, placental alkaline phosphatase reporter gene; SCL, stem cell leukaemia gene.

We next asked if LTR‐EC activity was restricted to the FL during development. The mouse embryonic liver develops at around stage E8 and ECs forming the microvasculature are first observed by stage E9‐E10, concomitant with the appearance of hematopoietic cells 62, 63. SCL‐PLAP^+^VE‐cad^+^CD45^−^ cells were detected by E10 (Fig. 4A). Independently of the developmental stage, most VE‐cad^+^CD45^−^ cells expressed CD31^+^, however Lyve1^+^ sinusoidal ECs only constituted 50 ± 4% of VE‐cad^+^CD45^−^ population by stage E10, progressively incrementing up to 90 ± 5% by stage E14 (Fig. 4B). To determine when LTR‐EC activity first appeared during FL development a number of total unfractionated E10 to E14 FL cells, (0.1‐5 × 10^6^ cells) (Table 2), containing equivalent numbers of SCL‐PLAP^+^VE‐cad^+^CD45^−^ cells (0.2‐1.1 × 10^4^ cells) were transplanted per recipient (Supporting Information Table 3). None of the 9 analyzed mice receiving E10 FL cells presented donor‐derived liver vascular engraftment, whereas a total of 15 out of 17 recipient mice receiving E11 to E14 FL cells showed SCL‐PLAP^+^ endothelial v.c. in the liver (Table 2). A substantial fraction of the chimeras with liver vascular engraftment also presented vascular clusters in the heart (7 positive out of 17 transplanted mice) (Supporting Information Table 4) and hemato/vascular clusters in the kidneys (not shown). The low SCL‐PLAP^+^VE‐cad^+^CD45^−^ cell numbers obtained and transplanted from E10 FL could explain the reduction of LTR‐EC activity. Alternatively, FL SCL‐PLAP^+^VE‐cad^+^CD45^−^ cells may acquire LTR‐EC activity as a maturational process along development, analogous to the maturational events driving the changes in the reconstitution potential of immature HSC to acquire HSC properties 50, 63. Indeed, at E10 a greater proportion of VE‐cad^+^CD45^−^ cells were negative for Lyve1 (Fig. 4B), a marker proposed to be associated to maturation of liver endothelial cells during development 58.

**Table 2:** Spatial‐temporal mapping of LTR‐EC activity during development

Donor cells			Hematopoietic chimerism		Vascular chimerism	
Tissue	Stage	Cell no x10^6^ (ee)	n^o^ PLAP^+^/ Total mice^a^	% PLAP^+^ (Range values)^b^	n^o^ PLAP^+^/ Total mice^c^	PLAP^+^ v.c.a. x10^−3^ cm^2^ (Range values)
FL	E10	0.1‐0.14	0/13	0	0/9	0
		(2‐4)				
	E11	0.25‐0.5	5/6	10.56 ± 6.72	6/6	1.21 ± 0.52
		(2‐4)		(4.9‐18)		(0.23‐1.69)
	E12	1.3	5/5	12.84 ± 9.64	5/5	1.45 ± 0.74
		(1)		(1.6‐27)		(0.18‐2.08)
	E14	5	6/6	6.11 ± 5.73	4/6	0.81 ± 0.22
		(0.2)		(1‐13)		(0.49‐1)
Adult liver		5	0/6	0	0/6	0
AGM	E12	0.55	10/12	7.35 ± 5.58	4/10	0.023 ± 0.036
		(2)		(0.7‐16.5)		(0.0012‐0.076)
Yolk sac	E12	1	5/10	20.36 ± 10.4	0/5	0
		(1)		(3.4‐30)		

Receptor mice were transferred with the indicated number of donor cells obtained from the different tissues derived from SCL‐3′Enh‐PLAP transgenics. Most animals were also cotransplanted with 1 × 10^6^ BM‐LacZ^+^ cells. Mice were analyzed at >3 months post‐transplant. SCL‐PLAP^+^ hematopoietic chimerism was determined in peripheral blood leukocytes by PCR‐PLAP/LacZ and flow cytometry.

a Only mice presenting PLAP and/or LacZ PCR signal in circulation are included in the study. The number of SCL‐PLAP^+^ chimeras related to the total analyzed mice is indicated.

b Mean values for % SCL‐PLAP^+^ cells in the circulation are shown.

c Vascular chimerism was determined by detection of SCL‐PLAP^+^ vascular clusters (v.c.) on liver sections. The number of animals presenting SCL‐PLAP^+^ v.c. from the total number of analysed mice is shown. The mean ± SD values of tissue area containing the SCL‐PLAP^+^ v.c. referred to the total tissue area analysed (PLAP^+^ v.c.a.) are indicated for each group (individual values in Supporting Information Table 4). Data was obtained from 2‐4 independent transplantation experiments for each tissue.

Abbreviations: ee, embryo equivalent; PLAP, placental alkaline phosphatase reporter gene.

We next analyzed the presence of LTR‐EC activity in the adult liver. Endothelial engraftment potential of adult LSECs, has been shown by transferring purified CD31^+^ LSECs or a LSEC enriched NPCs fraction (LSEC‐NPC) into preconditioned adult mice 26, 29, 32. Flow cytometry analysis indicated that 4 ± 2.6% of LSEC‐NPC cells were SCL‐PLAP^+^VE‐cad^low+^CD45^−^ (Fig. 4A), most expressing the endothelial markers Lyve1 and CD31 (Fig. 4B). Despite the presence of an incremented percentage of ECs compared to E12 FL cells, less EC cords/colonies were obtained in vitro from LSEC‐NPC cells (Fig. 4C). To exclude any effects promoted by other cells contained in the LSEC‐NPC fraction, sorted VE‐cad^+^CD45^−^Lyve1^+^ cells were examined. This analysis confirmed the lower in vitro EC activity of adult as compared to FL counterpart cells, indicating that fetal and adult Lyve1^+^ ECs possessed different proliferative/differentiation potential. To further evaluate LTR‐EC activity, 5 × 10^6^ LSEC‐NPCs cells were transplanted per recipient. Analysis of the host mice at 4 months revealed only very few SCL‐PLAP^+^ sinusoidal‐like cells in the livers from 2 out of 6 chimeras (Fig. 4D, Table 2). This result shows that adult liver LSEC‐NPCs cells lack an inherent potential to reconstitute the liver microvasculature when transferred i.v. to Bu‐conditioned newborns.

To further determine if LTR‐EC activity was detected in other locations presenting hematopoietic activity in E12 embryos, we performed flow cytometry analysis and transplantation assays with cells obtained from the yolk sac (YS) and from the aorta‐gonads‐mesonephros (AGM) region, both of which are known to contain SCL‐PLAP^+^ cells 39. Despite the presence of SCL‐PLAP^+^VE‐cad^+^CD45^−^, Lyve1^+^ cells in both sites (Supporting Information Fig. 4A, 4B) 57, vascular grafts were rare in AGM and absent from YS chimeras (Table 2, Supporting Information Fig. 4C, Supporting Information Table 3). These results suggest that the FL is the principal source of SCL‐PLAP^+^VE‐cad^+^CD45^−^, Lyve1^+^ cells with LTR‐EC potential.

### Analysis of FL Derived Vascular Endothelial Clusters Integration in the Liver of Young Mice. Implications for Vascular Graft Functionality

**Figure 5:**
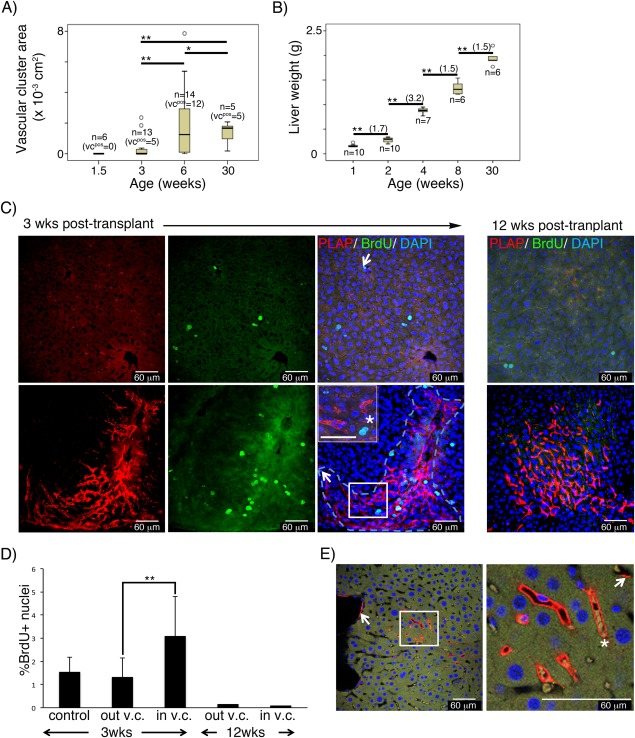
Functional integration of donor‐derived vascular graft in the liver at 3 weeks post‐transplantation. Livers from mice transplanted with E12 FL cells were analyzed at different times post‐transplant. **(A)**: Box plot representing the distribution and mean values of the SCL‐PLAP^+^ vascular cluster area (v.c.a.) at different ages. The total number of analyzed (*n*) and v.c. positive (v.c.^pos^) mice is indicated for each age group. Donor‐derived v.c. were first observed in 3 week‐old mice. The mean v.c.a. values were compared between groups using the U‐Mann–Whitney test (**, *p* < .05). Significant increment on v.c.a. are observed from 3 to 6 weeks post‐transplant. No significant v.c.a. size increment is observed at long term (from 6 to 30 weeks post‐transplant). **(B)**: Box plot representing the mean values of the liver weight at the different ages. Significant liver growth increment is observed at all ages (Student's *t* test, **, *p* < .05) with maximal weight increment from 2 to 4 weeks (fold increment in brackets). Mice were derived from 2 to 3 transplantation experiments for each age. **(C)**: Images from Z stacked optical liver sections from 3 and 12 weeks old FL chimeras, stained with antibodies anti‐PLAP, anti‐BrdU and DAPI. Representative images from liver regions without (top panel) and with (lower panel) SCL‐PLAP^+^ donor‐derived vascular clusters (v.c.) Most BrdU^+^ proliferating cells looked‐like hepatocytes (large size, single or multiple round nuclei, prominent nucleoli and moderate to low nucleus/cytoplasm ratio 66), (asterisk, in inset magnified field). Very few BrdU^+^ endothelial‐like cells are observed (arrows, elongated nuclei, vascular lumen location). **(D)**: Percentage of BrdU^+^ nuclei referred to the total DAPI^+^ nuclei determined in regions without v.c. (out v.c.) and within v.c. (in v.c.) as shown in (C). Number of fields to determine average %BrdU^+^ values: *n* = 12 from one 3weeks old control mouse; *n* = 14 from out‐v.c. and *n* = 10 from in‐v.c., from two 3 weeks old FL chimeras; *n* = 32 from out‐v.c. and *n* = 12 from in‐v.c., from two 12 weeks old FL chimeras. **, Unpaired two‐tailed Student's *t* test assuming *p* < .05; **(E)**: Optical confocal sections from 3D Z‐stacked confocal images showing PLAP^+^ endothelial cells forming part of large and micro‐vasculature (arrows) and forming whole functional donor derived vessels containing blood cells (asterisk) connected with circulation. Vessels from 7 out of 10 donor‐derived v.c. presented circulatory cells. Scale bars 60 μm. Abbreviation: PLAP, placental alkaline phosphatase reporter gene.

Functional integration of donor‐derived endothelium is a prerequisite for the recovery/modulation of the targeted organ. Functional endothelium should provide and respond to signals necessary for organ formation/regeneration, regulating tissue growth and cell proliferation. Coordinated growth of endothelium and tissue cell has been shown during liver organogenesis 64, regeneration 29, and damage recovery 65. To analyze functionality of the v.c. and whether v.c. associated with cell proliferation during neonatal liver growth we first determined the emergence and expansion of SCL‐PLAP^+^ v.c. Mice were transplanted with 10^6^ E12 FL cells and the frequency of vascular chimerism and vascular cluster areas (v.c.a.) assessed at different times post‐transplant. For these experiments we used unfractionated FL cells, as in these conditions the frequency of vascular chimeras was highly incremented compared with sorted populations (Tables 1 and 2). Sporadic donor‐derived cells were readily observed after a week without obvious SCL‐PLAP^+^ v.c. formation. Clusters were first detected at 3 weeks post‐transplantation (5 positive out of 13 transplanted mice; v.c.a range from positive mice, 0.28‐2.34 × 10^−3^ cm^2^) (Fig. 5A). Consistent increment on the frequency of vascular chimeras occurred by 6 weeks (12 positive out of 14 recipient mice) with significant v.c.a. growth compared to 3 weeks, (v.c.a range from positive mice 0.19‐7.86 × 10^−3^ cm^2^, Mann–Whitney test, *p* < .05). By 30 weeks, all mice presented vascular chimerism (5 positive out of 5 mice analyzed), reaching a v.c.a stabilization or even decrease (v.c.a. range 0.18‐2.18 x10^−3^ cm^2^) suggesting that vascular grafts have a limited expansion capability. During this period the liver presented a 1.7‐fold weight increment from 1 to 2 weeks old mice (0.16 ± 0.02 g to 0.28 ± 0.05 g) followed by a 3.2‐fold augmentation between 2 and 4 weeks (0,9 ± 0.04 g). Liver mass incremented at lower rates from 4 to 8 weeks (1.4 ± 0.1 g, 1.5‐fold weight increase) and from 8 to 30 weeks (1.9 ± 0.2 g, 1.5‐fold weight increase) (Fig. 5B). Thus, robust organ growth is observed between 2‐4 weeks, coincidental with v.c. expansion, and consequently proliferation activity is expected to occur.

To determine whether v.c. associated with cell proliferation activity within the 2‐4 age interval, livers were analyzed at a post‐transplantation time when v.c. are first detected and active liver growth occurs (3 weeks), and at later times when graft expansion stabilizes, liver growth slows down and minimal proliferation is expected (12 weeks). Thick liver sections were stained for BrdU/PLAP and 3D images analyzed using confocal microscopy. Proliferating BrdU^+^ cells were evident at 3 weeks and all SCL‐PLAP^+^ v.c. (*n* = 10) included BrdU^+^ cells (Fig. 5C). The majority of these cells appeared to be hepatocyte‐like cells. Moreover, the frequency of BrdU^+^ nuclei within the tissue area containing the v.c. was significantly higher (3.7 ± 1% BrdU^+^ nuclei) than in tissue areas without grafts (1.3 ± 0.2% BrdU^+^ nuclei), indicating a direct association of grafted cells and proliferating cell foci (Fig. 5D). At 12 weeks post‐transplantation no BrdU^+^ cells were observed within the v.c. (*n* = 10), indicating the absence of continuing proliferation in the grafted region. Based on these observations, we propose that the preferential association of expanding v.c. with proliferating hepatocyte‐like cells reflects functional integration of donor‐derived endothelium, reminiscent of sinusoidal liver endothelial cell activity that mediate proliferation during regenerative growth 29. At the moment we cannot distinguish if at 3 weeks post‐transplantation, expanding donor‐endothelium induces the proliferation of neighboring cells, or whether donor‐cells home into proliferative areas containing high levels of VEGF necessary for EC expansion. Indeed, both possibilities are not mutually exclusive.

Analysis of chimeras generated by transplantation with ubiquitously labeled SCL‐PLAP; act‐DsRed FL cells did not result in DsRed^+^ hepatocyte clusters (Supporting Information Fig. 5). This finding is consistent with previous results 35 and suggests that proliferating hepatocytes are not donor derived. Unexpectedly, very few BrdU^+^ nuclei among endothelial‐like cells were obtained and we also analyzed the expression of Ki67 and P‐H3 as G1/S/G2 and mitosis markers respectively. However, we found only very few proliferating liver endothelial cells. Most Ki67/P‐H3^+^ cells were Albumin^+^ hepatocytes (Supporting Information Fig. 6). It remains to be elucidated if mechanisms coordinating neonatal vascular graft/liver growth are similar to mechanisms acting during adult liver regeneration and whether endothelial expansion predominantly occurs through elongation/remodeling of the cell cytoplasm 67 or through proliferation identified by different markers 68.

Functionally grafted vasculature has to connect with the host vascular conduits to integrate into the transport network. The detection of administered fluorescent perfusant or the presence of circulatory cells within donor‐derived vasculature has been used to demonstrate functionality of vascular grafts 8, 24. 3D confocal image analysis from v.c. showed that circulating cells were present within the lumen of PLAP^+^ vessels at 3 weeks post‐transplant (circulating blood cells identified in vessels from 7 out of 10 v.c analyzed) (Fig. 5E), reinforcing the idea that donor‐derived vascular clusters represent functional vasculature perfused by circulation.

## Discussion

In the present study, using the expression of SCL‐3′Enh cis‐regulatory element in combination with transplantation and in vitro assays, we have isolated and characterized a novel progenitor population endowed with multiorgan long‐term reconstituting endothelial (LTR‐EC) potential from the E12 FL. Our previous studies have provided a comprehensive characterization of the hemato/vascular SCL‐3′Enh, establishing its activity pattern in a range of progenitor and mature cell types 39, 41, 69, 70. Here, we used the SCL‐3′Enh‐PLAP mouse model to reveal the lineage commitment and in vivo reconstituting potential of FL SCL‐PLAP^+^VE‐cad^+^CD45^−^cells. The identification of a new EC/EPC population with LTR‐EC potential using the SCL‐3′Enh expression vector, opens new avenues for manipulating vascular cells during organ development and in a transplantation context, potentially similar to the SCL‐3′Enh use for basic and translational studies of HSPCs 40, 71, 72, 73, 74, 75.

The FL SCL‐PLAP^+^VE‐cad^+^CD45^−^ population is heterogeneous, a fact that might be relevant for its function in transplantation. It is mainly composed by lineage committed sinusoidal type ECs that express different EC‐associated molecules, including the sinusoidal marker Lyve1 57, 58, 76, 77 and present EC activity in vitro. In addition, the SCL‐PLAP^+^VE‐cad^+^CD45^−^ population contains other minor Lyve1^−^ and/or KIT^+^ cell subsets endowed with in vitro B lymphoid cell and limited EC activity and their precise allocation within the hemato/endothelial hierarchy remains uncertain 49, 50, 61, 78. Taking into account that SCL‐PLAP^+^VE‐cad^+^CD45^−^ derived vascular grafts are not simultaneously detected in all analyzed organs, it is possible that SCL‐PLAP^+^VE‐cad^+^CD45^−^Lyve1^+^ cells are potentially true sinusoidal endothelial cells, predominantly contributing to liver endothelium. Conversely, the minor Lyve1^−^ and/or KIT^+^ cell subsets may contribute to a specific endothelial beds in the heart, kidney or lung and/or to the SCL‐PLAP^+^CD45^+^ hematopoietic cells identified in v.c. As in vitro assays do not always reflect in vivo potentials, additional cell‐surface markers, transcriptional profiling and transplantation assays of SCL‐PLAP^+^VE‐cad^+^CD45^−^Lyve1^+^ cells and Lyve1^−^ cells should allow to more precisely classifying FL cells with LTR‐EC potential.

SCL‐PLAP^+^VE‐cad^+^CD45^−^ cellular heterogeneity could also contribute to the generation and stabilization of the vascular graft within an organ via coordinated pro‐angiogenic and vasculogenic actions ascribed to different cell sub‐subsets, as proposed before for postnatal circulating/BM‐derived populations with neovascularization potential 3, 6. As we discuss below, our results suggest a potential role of transplanted FL HSCs and/or myeloid cells and their progeny as pro‐angiogenic cooperative cells, contributing to the increment of endothelial engraftment. Accordingly, transplantation of SCL‐PLAP^+^ 35 or SCL‐PLAP^+^VE‐cad^+^ cells, containing HSPCs 39 resulted in almost 100% of animals presenting liver vascular engraftment, while mice transplanted with SCL‐PLAP^+^CD45^−^ or SCL‐PLAP^+^VE‐cad^+^CD45^−^ populations, devoid of hematopoietic repopulation potential, yielded 23%‐33% of vascular chimerism. Taking into consideration that all analyzed host mice also presented LacZ^+^ adult BM‐derived hematopoietic chimerism, this suggested that a specific cooperative action from FL HSPCs and/or their hematopoietic cell progeny may constitute a key factor for achieving efficient EC/EPCs vascular reconstitution, potentially by exerting pro‐angiogenic activities, as no direct FL HSC‐derived EC contribution was observed. Distinctive characteristics of FL hematopoietic cells can be relevant for their putative enhanced pro‐angiogenic cooperative action. Compared to adult BM HSCs, counterpart FL cells present a more robust hematopoietic repopulation 79, 80, 81, increased migratory responses to chemotactic signals 82, enhanced trans‐endothelial migratory capacity, 83 and distinctive expression of Mac‐1 43, 84 and VE‐cad 47, 54, molecules potentially involved in their recruitment to sites of neovascularization 85, 86. Also, FL HSCs present a platelet/myeloid lineage‐skewed repopulation potential 81, 87, which can be a potentially relevant issue considering that myeloid cells constitute one of the main pro‐angiogenic hematopoietic cells recruited to sites of vascular damage 22, 88, 89, also involved in vessel anastomosis and remodeling during embryogenesis 90, 91. Potential cooperative pro‐angiogenic action of FL‐derived myeloid cells may be exerted at short‐term post‐transplantation, as no donor derived SCL‐PLAP^+^F4/80^+^ myeloid cells were observed in liver section from long‐term chimeras 35 (and data not shown). Further SCL‐PLAP^+^VE‐cad^+^CD45^−^ cells cotransplantation assays with different FL myeloid/macrophage cell subsets 91, 92 should clarify this issue.

Intrinsic characteristics of EC/EPC populations that are related to their origin and developmental stage may also account for vascular engraftment potential. Changes in liver endothelial cells during development have been associated with modulating marker expression, including Lyve1 58, 93. The microvascular sinusoids start forming in the FL around E9 of development and can be identified by the histological expression of Flk1, Stab1, and CD31. However, the hyaluronan‐receptor Lyve1 emerges in part of the sinusoids at E10, when sinusoidal lumen formation becomes more evident 58, 64, 93. In accordance with this data, we observed that while most E10 FL SCL‐PLAP^+^VE‐cad^+^CD45^−^ cells expressed CD31, only about 50% expressed Lyve1. However, by day E11 most SCL‐PLAP^+^VE‐cad^+^CD45^−^ cells were Lyve1^+^. As the LTR‐EC activity emerged around this time, acquisition of Lyve1 expression might be correlated with the appearance of LTR‐EC activity from the SCL‐PLAP^+^VE‐cad^+^CD45^−^ population. Acquisitions of other functional features, including engulfment capability and emergence of fenestrations, have also been associated to liver sinusoidal EC phenotypic changes along development 58, 93. However, the implications of Lyve1 expression on the function of EC/EPC remained speculative. In addition, Lyve1 knock‐out mice have no apparent liver phenotype 94, 95.

Differences between midgestation FL and adult liver EC/EPCs may account for the absence of reconstitution potential from the adult LSEC‐NPC cell fraction when transplanted into newborn mice. Adult liver‐derived LSECs and sinusoidal progenitor cells have been effectively used for correction of bleeding disorders 32 and promotion of liver regeneration 27, 29 in rodents and might thus be considered for therapeutic applications. However, vascular integration of transplanted adult LSECs requires extreme and highly damaging preconditioning regime of the host, including hepatectomy, treatment with toxic agents for ECs, irradiation or severe genetic predisposition 26, 28, 76, conditions that do not seem to be induced by the here applied busulfan treatment 96. FL Lyve1^+^ cells present features of immaturity, including the expression of progenitor associated marker CD34 and the lack of fenestrations 58. Interestingly, previous reports involving primary postnatal endothelial progenitors from the vascular wall have correlated the incremented in vitro OP9‐EC colony formation potential with an EC immature status 24. We also showed that FL Lyve1^+^ cells presented a higher EC cord/colony formation potential compared to adult counterpart cells, supporting the idea that FL cells are functionally more immature than adult LSEC cells. Moreover, comparative PCR‐based quantification of repopulation of monocrotaline treated mouse liver endothelium indicates that, related to adult liver, donor FL CD31^+^ ECs presented a more robust repopulation potential 97. This result suggests that FL Lyve1^+^ cells, likely responsible for liver vascular engraftment, possess characteristics of immature progenitor cells compared to their adult counterparts.

Although the use of primary or in vitro generated EC/EPC with FL‐EC characteristics could be regarded as a more efficient alternative to the use of adult LSEC, it is important to take into consideration that FL SCL‐PLAP^+^VE‐cad^+^CD45^−^ population reported here or human FL CD31^+^ population reported by others 97, might include hematopoietic cooperative cells modulating the effective vascular integration of endothelial lineage committed EC/EPCs. As promoting vasculogenesis is an essential step for fostering liver regeneration and repair 29, 98 or correcting coagulation defects 32, 76, it will be important to further investigate the characteristics and mechanisms that confer FL SCL‐PLAP^+^VE‐cad^+^CD45^−^, Lyve1^+^ population with engraftment advantages.

## Conclusion

Using the newborn transplantation model, we have characterized in the mouse fetal liver a unique SCL‐PLAP^+^VE‐cad^+^CD45^−^ population, mostly composed by Lyve1^+^ endothelial cells, that is endowed with multiorgan endothelial reconstitution potential (LTR‐EC activity). Considering clinical applications, FL SCL‐PLAP^+^VE‐cad^+^CD45^−^ cells may provide a more robust vascular engraftment than BM‐derived 35 or adult liver‐derived EC/EPC populations, constituting a novel and highly promising source of cells to study vascular reconstitution and repair in neonatal preclinical models and also might help guide the derivation of long term reconstituting vascular progenitors from pluripotent stem cells.

## Disclosure of Potential Conflicts of Interest

The authors indicate no potential conflicts of interest.

## Supporting information

Additional supporting information may be found in the online version of this article

Supporting Information

Supporting Information Tables.
