# Supplementary material for: Characterization of a Fetal Liver Cell Population Endowed with Long‐Term Multiorgan Endothelial Reconstitution Potential
Source: Stem Cells. 2016 Sep 28;35(2):507–21. doi: 10.1002/stem.2494 (PMC5298023; doi:10.1002/stem.2494)

**SUPPLEMENTAL MATRIALS AND METHODS**

**Cell preparation**

The FLs, aorta-gonads-mesonephros (AGM) regions and yolk sacs (YS) were removed in D-PBS Ca^+^Mg^+^ (Sigma‐Aldrich, USA) supplemented with 5% FCS (GE Helthcare, HyClone, USA) and 100U/ml penicillin, 100μg/ml streptomycin (1%P/S) (GE Helthcare, HyClone, USA). Cell suspension from pooled FLs was obtained by gentle pipetting. Cell suspension from pooled AGM and YS was obtained by incubation in 0.12% collagenase/D-PBS/10%FCS (Sigma‐Aldrich, USA) and gentle mechanical dispersion. Postnatal hematopoietic tissues (bone marrow, spleen, thymus and lymph nodes) were obtained by standard methods and mechanically homogenized. Blood was drawn from the tail and the heart of adult mice or the facial vein from young mice into PBS 0.3% EDTA and erythrocytes lysed as described, (<http://www.ahc.umn.edu/rar/facial_vein.html>).

**LSEC-NPC cell isolation from adult liver.**

Mice were anesthetized, the portal vein cannulized with a 26-gauge needle for perfusion and the posterior vena cava cut to allow outflow of the perfusion solution. Perfusion with Liver Perfusion Medium (Life Technologies, GIBCO, USA) at a flow rate of 5 ml/min for 10 min at 37ºC was followed by perfusion with Liver Digest Medium (Life Technologies, GIBCO, USA) for an additional 10 minutes. The liver was excised, cut and dissociated in Minimum Essential Medium α (MEMα, Life Technologies, GIBCO, UK). The cell suspension was passed through a 70 um nylon cell strainer, transferred into two 50 ml tubes and centrifuged (50g, 5min). Hepatocyte containing pellet (parenchymal cell fraction) was discarded and the supernatant collected and fractionated by Percoll (Sigma-Aldrich, USA) gradient centrifugation. Percoll gradients were prepared in 15-ml tubes with 2 ml stock Percoll solution at 75%, 1.5 ml at 35% and 1 ml of cell suspension in D-PBS placed at the top of the Percoll gradient. After centrifugation at 900g for 20 min, the lower band (between 35% and 75% interfaces, NPCs cell fraction) containing LSECs and hematopoietic cells was collected, washed and the pellet re-suspended in 10 ml PBS 5% FCS.

**Flow cytometry.**

The following antibodies were used: Anti-human Placental Alkaline Phosphatase (PLAP) from AbD, Serotec, UK; anti-CD45-PE/Biotin, -CD31-PE, -Kit-PE, -Flk1-PE, -CD34-PE, -Lyve-1-FITC/PE, -Mac-1-PE/Biotin, -CD19-PE, -CD4-FITC, and -CD8-FITC from Pharmingen, BD Biosciences (www.bdbiosciences.com); -VE-cadherin-AlexaFluor647/Biotin, from BioLegend, USA; -CD45-FITC, -B220-FITC, -Tie2-Biotin from e-Bioscience (www.ebioscience.com). Secondary antibody goat anti-rabbit AlexaFluor488 conjugated (Molecular Probes, www.probes.com) was used to visualize PLAP. PE-streptavidin (Pharmingen, BD Biosciences) and APC-streptavidin (BioLegend, USA) were used to visualize biotin-conjugated primary antibodies. Cell suspension was incubated with anti-Fcγ receptor antibody (anti CD16/CD32, BD Biosciences, USA) to reduce the non specific binding followed by incubation with specific antibodies and secondary reagents in PBS 5% FCS. 1 μg/ml of 7-Aminoactinomycine D (7AAD) (Sigma-Aldrich, USA) was added to exclude 7AAD^+^ dead cells from the analysis window. Cells were sorted at 4°C and collected in D-PBS 50%FCS 1%P/S, centrifuged and re-suspended in the appropriate medium. The purity of the sorted population was between 90% and 98%. Cell Quest and WinmDI2.8 software were employed for data analysis. Background levels and quadrants were established according to isotype controls staining, unstained cells and single-color staining on 7AAD^-^ gated cells from SCL-3’Enh-PLAP transgenics and wt controls. Isotype staining and unstained controls gave similar background levels. For most experiments unstained cells were used as controls.

**Newborn transplantation assays.**

Wild type pregnant females were injected intra peritoneal with 15.5 mg/kg of busulfan (Sigma-Aldrich) on the morning of days 17 and 18 of pregnancy to condition the newborn pups receptors for efficient donor cell engraftment. Pups were born on day 19 and cells injected into the facial vein on day 2 post-natal. Donor cells obtained from SCL-3’Enh-PLAP transgenics were transferred to the facial vein in 50 ul of D-PBS 1%FCS. Cells were co-injected with 10^6^ SCL-3’Enh-LacZ-transgenic bone marrow cells to monitor efficiently transplanted recipients as indicated by blood LacZ^+^ hematopoietic cell engraftment, instrumental when SCL-3’Enh-PLAP donor derived cells do not present circulating hematopoietic engraftment potential. In indicated experiments mice were transplanted with FL cells derived from double transgenic SCL-3’Enh-PLAP; actin-DsRed mice*.*

**Levels of hematopoietic chimerism by semi-quantitative PCR**

Chimerism was quantified by PCR for PLAP and LacZ on genomic DNA. Gel images from PCR products were obtained on a Typhoon 9410 scanner (GE Amersham Molecular Dynamics, USA) and comparison with PLAP and LacZ contribution controls determined using the Image Quant software (Molecular Dynamics, USA). The percentage of donor cell contribution was estimated using serial dilution controls of donor marker with unmarked DNA (100, 10, 1, 0.1and 0%) and Myo gene as the DNA normalization control. Normalized values were integrated into the curve obtained with serial dilutions of control genomic DNA.

**Histochemical detection of PLAP by NBT staining**

Organs were removed, zinc fixed (BD, Biosciences), cut into small fragments and paraffin-embedded. 5 μm thick non-serial microtome sections were generated. Slides were de-waxed, rehydrated and heat inactivated in PBS at 72^0^C to inhibit endogenous alkaline phosphatases (AP). Sections were incubated with AP-staining solution containing NBT and counterstained with brazilin (Anachem) or neutral red (Sigma-Aldrich). Dehydrated sections were mounted in DPX. Similar NBT-staining protocol was followed for 4% formaldehyde fixed cryopreserved tissues or 50 μm thick vibratome sections.

**Quantification of vascular engraftment on NBT stained liver sections.**

For estimation of the frequency of mice presenting vascular chimerism, 10-24 non-consecutive liver sections (average tissue area of 13.7±4.9 cm^2^ from >3 months old mice and 5-10 sections (average tissue area of 5.8±1.4 cm^2^) from <2 months old mice were NBT stained and PLAP^+^ vascular-like clusters scored. NBT positive signal is considered to form a vascular-like cluster if staining on micro-vascular endothelial-like cells is distributed over a tissue area including at least three hepatocytes (Sup. Figure 1C). For evaluation of the relative level of liver vascular engraftment, the tissue area within the PLAP^+^ vascular cluster was quantified and referred to the total tissue section area analyzed using the Arbitrary area tool from Image AnalySIS software program V3.1.110, (soft Imaging Systems GmbH, Germany, [http://www.soft-imaging.net](http://www.soft-imaging.net/)). The relative level of vascular engraftment for each individual was referred to 1 cm^2^ of the total tissue area analyzed and calculated as SCL-PLAP^+^ cluster area/total tissue area. Low magnification images were obtained with a Leica MZ7.5 stereoscope including a Nikon DS5 digital camera. Higher magnification bright field images were obtained with Leica DM5000B microscope and a Leica DC-180 digital camera.

**Antibodies and Immuno-histofluorescence staining.**

Antibodies were as follows: rabbit anti-human PLAP (1:100), anti-CD31 (1:20), anti-CD45 and anti-CD45 Biotinylated (1:100), isolectin B4 biotinylated (IsoB4) (20 μg/ml, Sigma-Aldrich, USA), rat anti‐BrdU, (1:500, AbD SEROTEC, Germany), mouse anti-Ki-67 (1:100, BD Pharmingen, USA), rabbit anti-Phospho-Histone H3 (1:200, Sigma-Aldrich, USA), rabbit anti-Albumin Biotinylated (1:100, Accurate Chemical, Westbury, NY, USA). The following secondary antibodies were used: Cy5-conjugated streptavidin (1:500; Jackson Immunoresearch Laboratories, [http://www.jacksonimmuno.com](http://www.jacksonimmuno.com/)), goat anti-rabbit AlexaFluor488/568 conjugated, and goat anti-rat AlexaFluor488/647 conjugated (1:500; Invitrogen, Molecular Probes, USA). For detection of PLAP-donor cell contribution to liver, heart, kidney and lung, 5 μm sections from zinc-fixed and paraffin-embedded tissues were re-hydrated, blocked by 2 h treatment with PBS/20% goat serum (GS) and incubated overnight at 4°C with primary antibodies anti-PLAP and anti-CD45 followed by secondary antibodies. Then, IsoB4-biotin was incubated overnight followed by and Cy5-streptavidin. The procedure was modified for combinations of antibodies anti-PLAP, anti-CD45biotin and anti-CD31, incubating first with anti-PLAP and anti-CD31, followed by secondary antibodies, fixation with 4% formaldehyde for 10 minutes and incubation with anti-CD45 biotin. Cy5-streptavidin was used to reveal CD45 expression. Similar staining protocols were used for detection of DsRed and PLAP on cryo-sections generated from 4% formaldehyde-fixed tissue, cryopreserved by serial incubation in PBS 30% sucrose solution followed by immersion in OCT (Tissue-Tek, NL) and snap frozen on dry ice. For detection of Ki67, P-H3 and albumin markers, sections from 4% formaldehyde-fixed and paraffin-embedded tissues were treated with Declere (Cell Marque, USA) for antigen retrieval according to the manufacturer instructions, blocked with PBS/10%GS/0.1%Triton solution and incubated with primary and secondary antibodies as indicated. PLAP reporter could not be detected after antigen retrieval. Slides were mounted in Farmount medium (Dako, USA-Aldrich, USA) with 0.5 μg/ml DAPI (Sigma). Immunofluorescence images were obtained with a Leica DM2500/TCS SPE (Leica Microsystems, http://www.leica-microsystems) confocal microscope using a LAS AF software equipped with a 488, 532, and 635 lasers.

***CD31 staining of endothelial cord/colonies.***

After 10 minutes fixation in 5% methanol/DMSO, cells were washed with PBS, incubated with 0.3%H_2_O_2_/methanol for 30 min followed by 2 times wash in PBT (PBS/0.05%Tween-20) and incubation with blocking solution (3% powdered fat free milk in PBS). Cells were then incubated with primary anti-CD31 antibody (1:200) at 4ºC overnight followed by incubation with a secondary anti-rat biotinilated antibody (1:200) at room temperature for 60 min. CD31 staining was revealed by 30 min incubation with the ExtrAvidin®−Peroxidase conjugate (1:400, Sigma-Aldrich, Israel) followed by adding diaminobenzidine substrate (DAB, peroxidase kit, VECTOR, USA). Each colony was identified as containing a central flat body from which one or more cord-like structures irradiated.

**BrdU immuno-detection.**

Livers were fixed in 4% formaldehyde solution for 3 days at 4°C, transferred to a 15% glucose 2% formaldehyde solution buffer for 24 hours and 70 μm thick vibrotome (Leica, Wetzlar) sections collected in PBS and placed in 24 well plates. For BrdU/PLAP co-staining, sections were incubated with 0.21% citrate buffer at 95ºC for antigen retrieval followed by 2 hours treatment with blocking solution (PBS/3% bovine serum albumin/1% Tween®20) and incubation with antibodies anti-BrdU and anti-PLAP and DAPI. The number of total nuclei per image was determined using the Spot function on the Imaris software (Version 7.6.3) and the number of BrdU-positive nuclei counted manually. PLAP-positive cluster areas were outlined with the Surface function, Imaris.


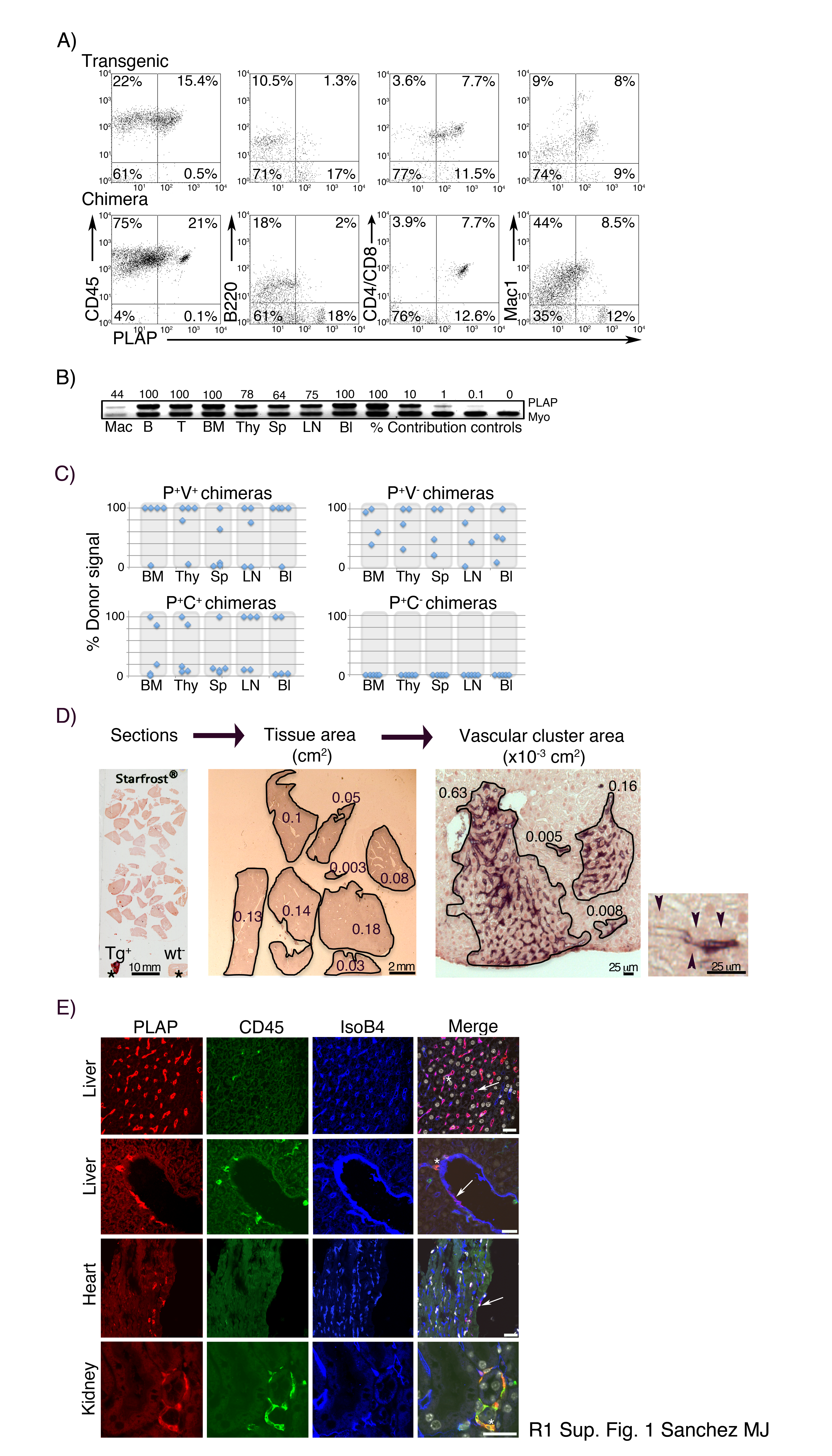


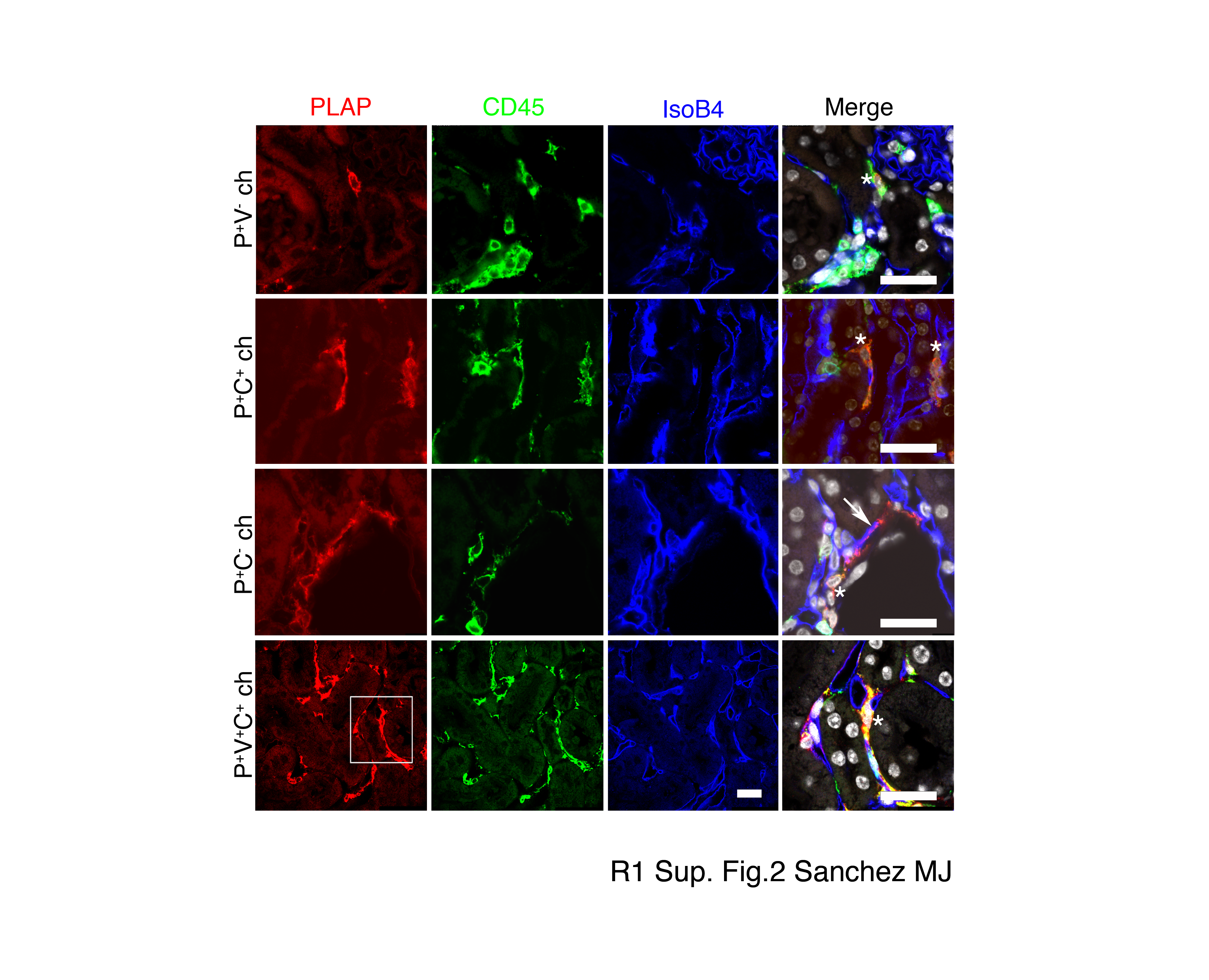


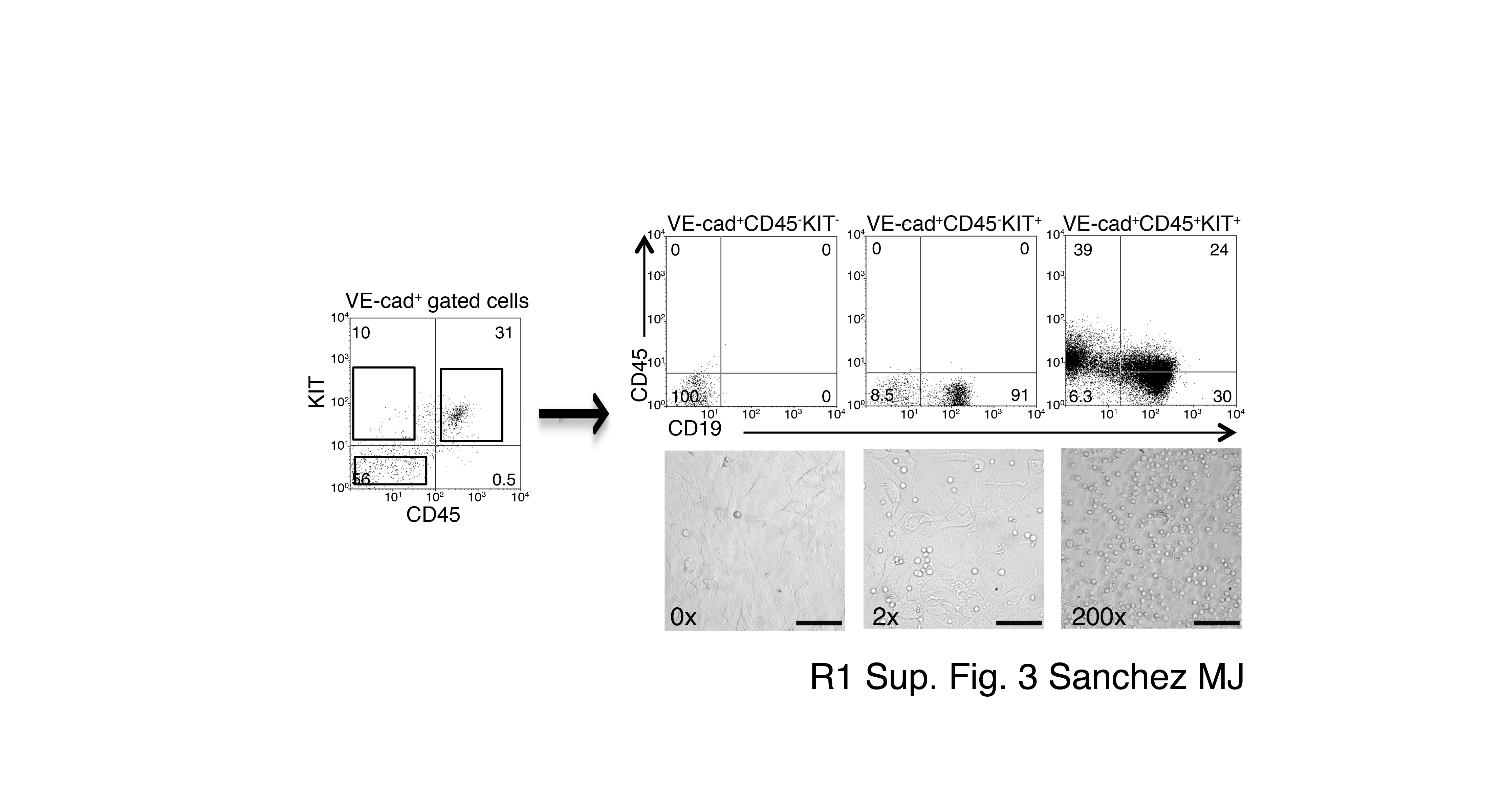


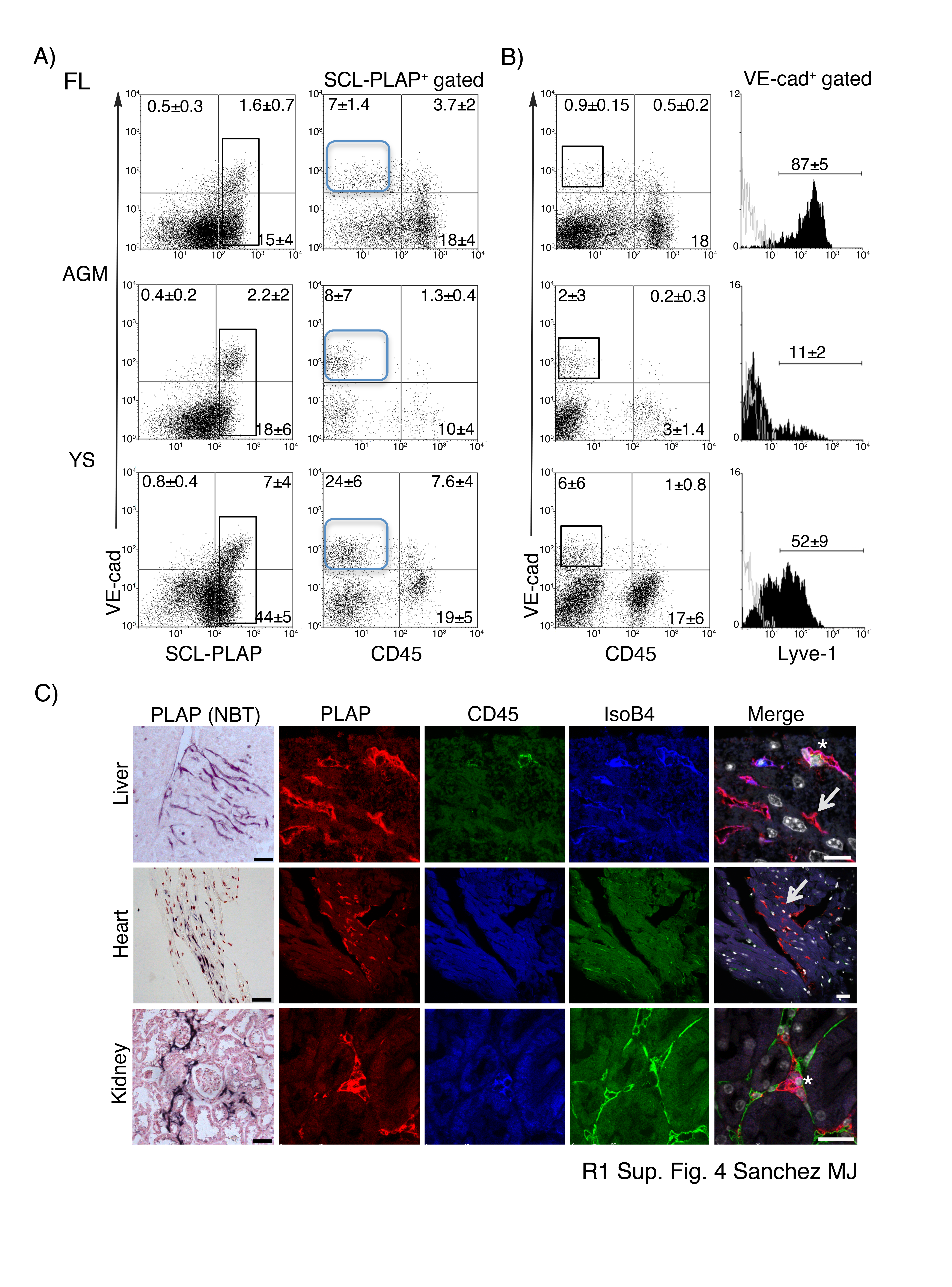


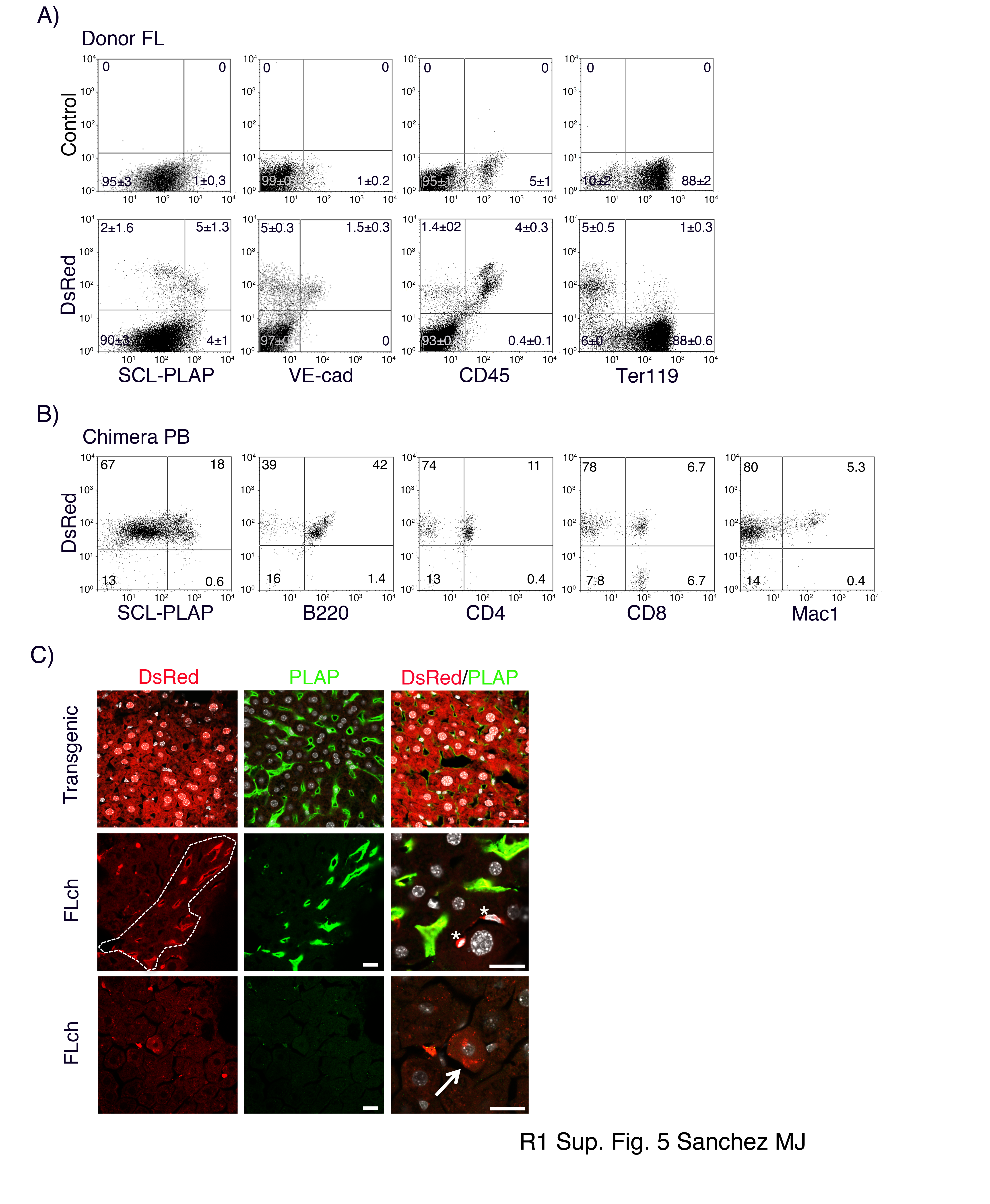


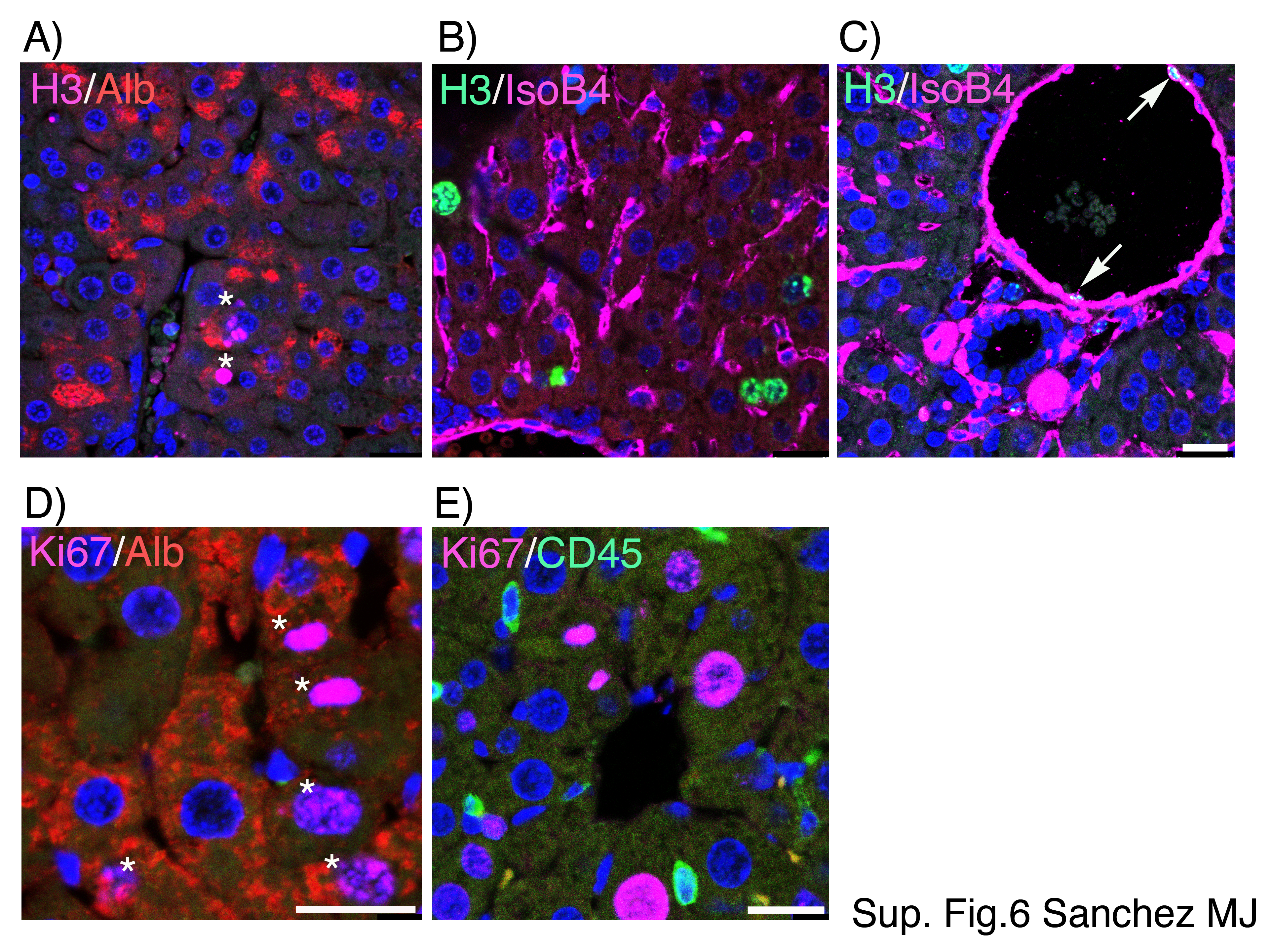

Supplement: Supplementary file 1 — Supporting Information [file STEM-35-507-s001.doc]
