# Supplementary material for: Characterization of a Fetal Liver Cell Population Endowed with Long‐Term Multiorgan Endothelial Reconstitution Potential
Source: Stem Cells. 2016 Sep 28;35(2):507–21. doi: 10.1002/stem.2494 (PMC5298023; doi:10.1002/stem.2494)
Supplement: Supplementary file 2 — Supporting Information Tables. [file STEM-35-507-s002.docx]

**SUPPLEMENTAL TABLES**

**Sup. Table 1**. LTR-EC activity from FL SCL-PLAP^+^ cell populations.

| Transplanted  cell population | Mice ID | %PLAP in circulation | Vascular chimerism | | | | |
| --- | --- | --- | --- | --- | --- | --- | --- |
|  |  |  | Liver | | | Heart | Kidney |
|  |  |  | Area analysed  (cm^2^) | Total PLAP^+^ cluster area  (x10^-3^ cm^2^ ) | Relative PLAP^+^ cluster area  (x10^-3^ cm^2^) |  |  |
| VE-cad^+^ | 5796 | 11 | 68.4 | 0 | 0 | ND | ND |
|  | 5800 | 14 | 60.9 | 0.23 | 0.004 | - | ++ |
|  | 6132 | 1 | 9.8 | 0. 40 | 0.041 | + | ++ |
|  | 6151* | 11 | 9.8 | 5.21 | 0.532 | +++ | ++ |
|  | 6152* | 8 | 9,8 | 0.92 | 0.093 | + | ++ |
|  | 6153 | 5 | 4.9 | 0 | 0 | ND | ND |
|  | 7050* | 9 | 9.8 | 10.25 | 1.045 | +++ | +++ |
|  | 7051 | 3 | 9.8 | 8.05 | 0.821 | - | ND |
| VE-cad^-^ | 5935 | 2.3 | 27.6 | 0 | 0 | **-** | + |
|  | 5936* | 10.8 | 25.7 | 0 | 0 | **-** | + |
|  | 7053 | 1 | 11.0 | 0 | 0 | **-** | + |
|  | 7056 | 2.5 | 9.8 | 0 | 0 | **-** | ND |
|  | 8012* | 16 | 9.8 | 0 | 0 | ND | ++ |
|  | 8013* | 14 | 9.8 | 0 | 0 | ND | + |
|  | 8009 | 22 | 9.8 | 0 | 0 | ND | ++ |
| CD45^+^ | 4725 | 5 | 4.9 | 0 | 0 | ND | ND |
|  | 4727 | 2.6 | 2.6 | 0 | 0 | **-** | + |
|  | 4892 | 1 | 1.7 | 0 | 0 | ND | ND |
|  | 5255 | 8 | 8.7 | 0 | 0 | ND | ND |
|  | 5256* | 17 | 13.2 | 0 | 0 | **-** | + |
|  | 5313* | 8 | 8.4 | 0 | 0 | **-** | + |
|  | 5314* | 12 | 12 | 0 | 0 | **-** | ++ |
|  | 5343 | 15 | 15.3 | 0 | 0 | **-** | ++ |
|  | 5344 | 12 | 12.8 | 0 | 0 | - | ND |
|  | 5464 | 3.4 | 34.5 | 0 | 0 | ND | ND |
|  | 5465 | 1 | 1 | 0 | 0 | ND | ND |
|  | 5466 | 6 | 5.7 | 0 | 0 | ND | ND |
|  | 6724 | 10 | 9.9 | 0 | 0 | ND | ND |
|  | 6739 | 6 | 6.2 | 0 | 0 | ND | ND |
|  | 6988 | 5 | 4.7 | 0 | 0 | ND | ND |
|  | 6989 | 4 | 4.4 | 0 | 0 | ND | ND |
|  | 6985 | 12 | 11.8 | 0 | 0 | ND | ND |
|  | 6986 | 9 | 8.7 | 0 | 0 | ND | ND |
|  | 6987 | 8 | 7.9 | 0 | 0 | ND | ND |
| CD45^-^ | 5296 | <0.1 | 25.7 | 0 | 0 | ND | ND |
|  | 5298* | <0.1 | 13.9 | 0.264 | 0.190 | - | - |
|  | 5342 | <0.1 | 26.2 | 0 | 0 | ND | ND |
|  | 5462 | <0.1 | 17.2 | 0 | 0 | ND | ND |
|  | 5461 | <0.1 | 4.9 | 0 | 0 | ND | ND |
|  | 6740* | <0.1 | 11.0 | 0.104 | 0.094 | - | + |
|  | 6733 | <0.1 | 9.8 | 0 | 0 | ND | ND |
|  | 6734 | <0.1 | 9.8 | 0 | 0 | ND | ND |
|  | 6735 | <0.1 | 9.8 | 0 | 0 | ND | ND |
|  | 6736 | <0.1 | 9.8 | 0 | 0 | ND | ND |
|  | 6975 | <0.1 | 11.0 | 0 | 0 | ND | ND |
|  | 6976 | <0.1 | 9.8 | 0 | 0 | ND | ND |
|  | 6977* | <0.1 | 10.4 | 0.193 | 0.185 | - | + |
| VE-cad^+^CD45^+^ | 8007 | PCR+ | 19.6 | 0 | 0 | ND | ND |
|  | 8014* | 8 | 19.6 | 0 | 0 | - | +++ |
|  | 8224* | 17 | 9.8 | 0 | 0 | - | +++ |
|  | 8227* | 14 | 9.8 | 0 | 0 | - | +++ |
|  | 8228 | 15 | 9.8 | 0 | 0 | - | +++ |
|  | 8229* | 10 | 9.8 | 0 | 0 | ND | +++ |
|  | 8962 | PCR+ | 12.2 | 0 | 0 | - | +++ |
|  | 8499-1 | 19 | 12.2 | 0 | 0 | ND | +++ |
|  | 8499-2 | 14 | 12.2 | 0 | 0 | ND | +++ |
| VE-cad^+^CD45^-^ | 8018 | <0.1 | 12.2 | 0 | 0 | +++ | - |
|  | 8020 | <0.1 | 12.2 | 0 | 0 | - | - |
|  | 8021* | <0.1 | 12.2 | 2.2 | 0.1803 | +++ | - |
|  | 8958 | <0.1 | 12.2 | 0 | 0 | - | - |
|  | 8959 | <0.1 | 12.2 | 0 | 0 | - | - |
|  | 8499* | <0.1 | 1.1 | 31 | 27.43 | +++ | +++ |
|  | 11099 | <0.1 | 12.2 | 0 | 0 | - | - |
|  | 11100* | <0.1 | 2.4 | 11,7 | 4.77 | - | + |
|  | 11101* | <0.1 | 12.2 | 0 | 0 | + | + |

Indicated cell sorted populations derived from E12 FL SCL-3’Enh-PLAP transgenics were transferred together with BM-LacZ^+^ cells to newborn mice and engraftment analysis performed at 3-8 months. Listed mice ID presenting PLAP and/or LacZ PCR signal in circulation are indicated. The % of SCL-PLAP^+^ cells in circulation is determined by flow cytometry. Quantification of the tissue area analysed for endothelial engraftment was performed on NBT stained liver sections (Sup. Figure 1D). Total tissue area, tissue area containing the SCL-PLAP^+^ vascular-like clusters and the relative tissue area containing the SCL-PLAP^+^ vascular-like clusters referred to the total tissue area analysed are indicated for each mouse. For heart and kidney 5 to 10 non consecutive sections were stain for NBT and scored for donor signal as follows: -, no signal; +, sporadic signal; ++, abundant disperse signal; +++, abundant signal and detection of SCL-PLAP^+^ vascular-like clusters (as represented in Figure 2A). Triple immunostaining for detection of PLAP, CD45 and IsoB4 indicated that the heart clusters from SCL-PLAP^+^VE-cad^+^ (n=2) and SCL-PLAP^+^VE-cad^+^CD45^-^ (n=3) chimeras are formed by SCL-PLAP^+^CD45^-^IsoB4^+^ endothelial cells, (Sup. Figure 1E and Figure 2B). (*) The kidneys from indicated SCL-PLAP^+^VE-cad^+^ (n=3), SCL-PLAP^+^VE-cad^-^ (n=3), SCL-PLAP^+^CD45^+^ (n=3) and SCL-PLAP^+^Ve-cad^+^CD45^+^ (n=4) chimeras were immunostained and all presented donor derived SCL-PLAP^+^CD45^+^ blood cells. Endothelial cell contribution in the kidney was only observed in SCL-PLAP^+^CD45^-^ and SCL-PLAP^+^VE-cad^+^CD45^-^ chimeras (Sup. Figure 2 and Figure 2B). ND, not done.

**Sup. Table 2**. Characterization of the hemato/endothelial nature of donor-derived SCL-PLAP^+^ cells located in large liver vessels from SCL-PLAP^+^VE-cad^+^CD45^+^ long term chimeras.

| Type of chimera | Circulation | Liver Sections | | | | | | | |
| --- | --- | --- | --- | --- | --- | --- | --- | --- | --- |
|  |  | nº of large vessels with PLAP^+^ cells | | | nº of individual PLAP^+^ cells analysed | | | | |
|  |  |  | | | Phenotype of cells within the intima/luminal area | | | | |
|  | % PLAP^+^ | Total | Positive | % | Total | CD45^-^IsoB^-^ | CD45^-^ IsoB^+^ | CD45^+^ IsoB^-^ | CD45^+^ IsoB^+^ |
| P^+^V^+^C^+^ |  |  |  |  |  |  |  |  |  |
| ch 8224 | 17 | 232 | 95 | 41 | 15 | 0 | 0 | 10 | 5 |
| ch 8229 | 10 | 97 | 49 | 50 | 5 | 0 | 0 | 3 | 2 |
| P^+^V^+^C^-^ |  |  |  |  |  |  |  |  |  |
| ch 11100 | 0 | 202 | 10* | 5 | 10 | 1 | 9 | 0 | 0 |

Livers from the indicated chimeras, presenting similar levels of circulating SCL-PLAP^+^ cells were immunostained for PLAP, CD45 and IsoB4. The numbers of total medium-large vessels, as shown in Figure 1F, were scored from 3-5 sections. Vessels presenting SCL-PLAP^+^ cells were considered as positive and the % calculated. A total number of selected cells located in the intima/luminal area of the vessels (as in Figure 1F) were analysed by confocal Z-stack projections and co-localization of the indicated markers evaluated assigning the indicated phenotypes. No SCL-PLAP^+^CD45^-^IsB4^+^ endothelial cells were identified in SCL-PLAP^+^VE-cad^+^CD45^+^ (P^+^V^+^C^+^) chimeras whereas in SCL-PLAP^+^VE-cad^+^CD45^-^ (P^+^V^+^C^-^) chimeras almost all SCL-PLAP marked donor-derived cells within the luminal area of large vessels were SCL-PLAP^+^CD45^-^IsB4^+^ endothelial cells (Figure 1E). *large vasculature forming part of sinusoidal vascular clusters in most cases.

**Sup. Table 3.** Frequency of SCL-PLAP^+^VE-cad^+^CD45^-^ cells during development.

| Tissue | Stage | % SCL-PLAP^+^ | %SCL-PLAP^+^VE-cad^+^CD45^-^ | | Equivalent nº of SCL-PLAP^+^VE-cad^+^CD45^-^ transplanted cells per receptor (x10^4^) |
| --- | --- | --- | --- | --- | --- |
| FL | E10 | 40±9 | 2.8±1.1 | | 0.2±0.1  1±0.2  1.1±0.1  0.95±0.7  19±13  1.2±1.6  (0.05 – 3.9)  5.5±6  (1.1 – 10) |
|  | E11 | 66±3 | 2.6±0.8 | |  |
|  | E12 | 17±6 | 0.9±0.1 | |  |
|  | E14 | 3.7±3 | 0.2±0.1 | |  |
| AL |  | 21±9 | 4±2.6 | |  |
| AGM | E12 | 18±10 | 2±3 | |  |
|  |  |  | (0.1 – 7.8) | |  |
| Yolk Sac | E12 | 52±12 | 6±6 | |  |
|  |  |  | (1.1 – 10) | |  |
|  | | | |  | |

Cell suspension was prepared from pools of the indicated tissues obtained at different developmental stage from SCL-3’Enh-PLAP transgenics. For adult liver (AL) cell suspension was prepared from individual livers. Cells were stained with antibodies for PLAP, VE-cad and CD45 and analysed by flow cytometry. Representative analysis windows and quadrants are shown in Figure 4. The equivalent nº of transplanted SCL-PLAP^+^VE-cad^+^CD45^-^ cells are extrapolated from the total number of transplanted cells shown in Table 2. Mean values and the SD are indicated. Range values are shown in brackets. Values obtained from at least 4 adult individuals and from pooled embryonic/fetal tissues obtained from at least 3 independent litters for each stage.

**Sup. Table 4**. Spatial-temporal mapping of LTR-EC activity.

| Transplanted  cells | Mice ID | Vascular chimerism | | | |
| --- | --- | --- | --- | --- | --- |
|  |  | Liver | | | Heart |
|  |  | Area analysed  (cm^2^) | Total PLAP^+^ cluster area  (x10^-3^ cm^2^) | Relative PLAP^+^  cluster area  (x10^-3^ cm^2^) |  |
| E10 FL | 8001 | 9.80 | 0 | 0 | ND |
|  | 8002 | 9.80 | 0 | 0 | ND |
|  | 8198 | 9.80 | 0 | 0 | ND |
|  | 8192 | 9.80 | 0 | 0 | ND |
|  | 8193 | 9.80 | 0 | 0 | ND |
|  | 8194 | 9.80 | 0 | 0 | ND |
|  | 8353 | 9.80 | 0 | 0 | ND |
|  | 8354 | 9.80 | 0 | 0 | ND |
|  | 8355 | 9.80 | 0 | 0 | ND |
| E11 FL | 7041 | 9.80 | 12.79 | 1.30 | +++ |
|  | 7046 | 9.80 | 10.88 | 1.10 | - |
|  | 7195 | 11.03 | 2.58 | 0.23 | - |
|  | 7198 | 10.42 | 15.35 | 1.47 | - |
|  | 7728 | 9.80 | 16.66 | 1.69 | - |
|  | 7729 | 9.80 | 14.62 | 1.49 | +++ |
| E12 FL | 6726 | 8.65 | 15.62 | 1.80 | +++ |
|  | 6729 | 5.04 | 0.90 | 0.18 | +++ |
|  | 6731 | 5.30 | 8.86 | 1.67 | ND |
|  | 6805 | 5.22 | 10,88 | 2.08 | +++ |
|  | 6806 | 4.10 | 6.38 | 1.55 | - |
| E14 FL | 6551 | 5.49 | 5.51 | 1.00 | +++ |
|  | 6552 | 6.58 | 5.44 | 0.82 | +++ |
|  | 6553 | 7.37 | 0 | 0 | - |
|  | 6554 | 4.80 | 2.38 | 0.49 | ND |
|  | 6555 | 3.50 | 3.40 | 0.95 | - |
|  | 6556 | 5.43 | 0 | 0 | ND |
| Adult liver | 10649 | 9.80 | 0 | 0 | ND |
|  | 10650 | 9.80 | 0 | 0 | ND |
|  | 10651 | 9.80 | 0 | 0 | ND |
|  | 10748 | 9.80 | 0 | 0 | ND |
|  | 10750 | 9.80 | 0 | 0 | ND |
|  | 10751 | 9.80 | 0 | 0 | ND |
| E12 AGM | 5815 | 16.07 | 0.02 | 0.0012 | +++ |
|  | 5816 | 38.32 | 0.31 | 0.008 | +++ |
|  | 5817 | 19.61 | 0 | 0 | ND |
|  | 5819 | 37.60 | 0 | 0 | ND |
|  | 5820 | 14.48 | 0.09 | 0.0057 | - |
|  | 5821 | 12.52 | 0 | 0 | ND |
|  | 5822 | 11.20 | 0 | 0 | ND |
|  | 5823 | 10.02 | 0 | 0 | ND |
|  | 6096 | 10.42 | 0 | 0 | ND |
|  | 6774 | 19.61 | 1.5 | 0.0766 | +++ |
| E12 YS | 5805 | 27.94 | 0 | 0 | ND |
|  | 5806 | 15.25 | 0 | 0 | ND |
|  | 5808 | 11.03 | 0 | 0 | ND |
|  | 5810 | 13.56 | 0 | 0 | ND |
|  | 6777 | 9.80 | 0 | 0 | ND |

Cells were prepared from indicated tissues derived from pooled SCL-3’Enh-PLAP transgenic embryos and individual adult livers and transferred to newborn mice. Most animals were also co-transplanted with BM-LacZ^+^ cells (as indicated in Table 2). Only mice presenting PLAP and/or LacZ PCR signal in circulation are included in the study and identified by the I.D. number. Vascular chimerism was determined by NBT staining for detection of SCL-PLAP^+^ vascular-like clusters on liver sections at > 3 months post-transplant. Quantification of vascular cluster area was performed as indicated in Sup Table 1. The presence of heart vascular-like clusters was assessed by NBT staining in some of the mice presenting SCL-PLAP^+^ vascular clusters in the liver and scored as indicated in Sup Table 1. Triple immunostaining for detection of PLAP, CD45 and IsoB4 was performed on heart sections showing that donor-derived cells are SCL-PLAP^+^CD45^-^IsoB4^+^ endothelial cells (Sup. Figure 4C). Immunostaining was also performed on kidneys from E11 FL (n=3) and AGM (n=2) chimeras. Kidney SCL-PLAP^+^ clusters mostly contained SCL-PLAP^+^CD45^+^IsoB5^-^ blood and SCL-PLAP^+^CD45^-^IsoB5^-^ interstitial cells. SCL-PLAP^+^CD45^-^IsoB5^+^ endothelial cells were not detected (Sup. Figure 4C).
